# Acute experimental colitis in 5xFAD Alzheimer's disease mice leads to enhanced monocyte infiltration into the brain accompanied by reduced β‐amyloid deposition

**DOI:** 10.1002/alz.70292

**Published:** 2025-06-02

**Authors:** Nanda K. Navalpur Shanmugam, Frank Zamudio, Deepak K. Vijaya Kumar, Kianna A. Barrett, Ryan VanDoren, Meng Chen, Olivia M. Barr, Sara Watson, Chih‐Chung Jerry Lin, William A. Eimer, Mehdi Jorfi, Se Hoon Choi, Robert D. Moir, Rudolph E. Tanzi

**Affiliations:** ^1^ Genetics and Aging Research Unit Henry and Allison McCance Center for Brain Health Department of Neurology Massachusetts General Hospital, and Harvard Medical School Charlestown Massachusetts USA

**Keywords:** colitis, intestinal inflammation, neuroinflammation, β‐amyloid

## Abstract

**INTRODUCTION:**

Emerging evidence has connected Alzheimer's disease (AD) to systemic inflammation, intestinal abnormalities, and altered gut microbiota, highlighting the significance of the gut–brain axis. Here, we investigated the impact of acute experimental colitis (acute colitis) on AD pathology.

**METHODS:**

Acute colitis was induced in 2‐month‐old 5xFAD mice using dextran sodium sulfate (DSS) to assess the effects of intestinal inflammation on the microbiome, systemic inflammation, neuroinflammation, and beta‐amyloid deposition.

**RESULTS:**

Induction of acute colitis in 5xFAD mice led to microbial dysbiosis and systemic inflammation. As a result, monocyte infiltration was observed in the brain accompanied by reduced cerebral beta‐amyloid deposition and increased beta‐amyloid efflux into the bloodstream.

**DISCUSSION:**

Increased infiltration of monocytes and elevated beta‐amyloid release into the bloodstream could both be responsible for the reduced beta‐amyloid deposition in 5xFAD mice following acute colitis. These results further highlight an important connection between gut‐induced peripheral inflammation and the progression of AD.

**Highlights:**

- Microbial dysbiosis occurs as a result of acute colitis in 5xFAD mice.
- Acute colitis in 5xFAD mice affects beta‐amyloid deposition.
- Increased IL‐2 and IL‐6 cytokine levels in the hippocampus of 5xFAD colitis mice.
- Colitis in 5xFAD mice increases serum proinflammatory cytokine levels and endotoxins.
- Acute colitis in 5xFAD mice increases monocyte infiltration and serum beta‐amyloid.

## 1 BACKGROUND

Alzheimer's disease (AD) patients suffer not only from neurodegeneration and cognitive impairment, but also from gastrointestinal complications owing to stress, medication, antibiotics, or a change in diet.^1^, ^2^ As a result, the past decade has witnessed a surge in research on the influence of the gut microbiome on AD pathogenesis which found that beta‐amyloid and tau burden associate with decreased gut microbial diversity in preclinical AD individuals (enteric dysbiosis or gut dysbiosis), suggesting altered gut‐brain signaling in AD.^2^, ^3^ Leaky gut syndrome (LGS) is caused by extended enteric dysbiosis that weakens the intestinal walls. The resulting intestinal perforations could permit the entry of microbes and microbial products into circulation, thereby causing systemic inflammation throughout the body, including the brain, which could result in neuropathology.^4^, ^5^, ^6^ Mounting evidence links an abnormal increase in specific enteric microbes to a range of chronic late‐life inflammatory diseases whose primary pathologies lie outside the gastrointestinal (GI) tract.^7^, ^8^ Consistent with these reports, 70% of AD patients were shown to have increased levels of fecal and cerebrospinal fluid (CSF) calprotectin, an intestinal inflammatory marker that correlated with greater beta‐amyloid (beta‐amyloid) burden, supporting the link between intestinal inflammation and AD pathology.^9^, ^10^ Intestinal inflammation could contribute to the systemic inflammation widely reported in the literature, such as the meta‐analysis of 175 studies, including 13,344 AD subjects, reporting elevated pro‐inflammatory markers tumor necrosis factor α (TNFα), interleukin (IL)‐2, IL‐6, IL‐8, interferon‐ γ, and C‐reactive protein compared to healthy controls.^11^, ^12^ Collectively, these observations suggest that enteric dysbiosis plays an important role in diseases associated with aging, possibly initiating a cascade of events starting with intestinal inflammation (due to dysbiosis), followed by systemic inflammation, and perhaps, neurodegeneration and cognitive impairment.

To determine whether intestinal inflammation directly impacts AD neuropathology, here, we chemically induced acute experimental colitis (acute colitis) using dextran sodium sulfate (DSS) in 2‐month‐old 5xFAD mice. Then we assessed microbiome alterations, systemic inflammation, neuroinflammation, and beta‐amyloid deposition. We found that acute colitis in 5xFAD mice reduced beta‐amyloid deposition in the brain while increasing serum beta‐amyloid levels, signifying increased efflux across the blood–brain barrier (BBB). These effects were associated with altered microbial diversity, increased neuroinflammation, and pro‐inflammatory cytokine signaling in the brain. We also observed enhanced monocyte and neutrophil infiltration into the brain. Ultimately, our collective findings suggest that intestinal inflammation could promote divergent effects, such as increased efflux of beta‐amyloid into the bloodstream or possibly increased phagocytosis by infiltrating leukocytes (particularly monocytes), thereby reducing beta‐amyloid deposition in the brain. Also, since beta‐amyloid is an antimicrobial peptide (AMP),^13^, ^14^, ^15^, ^16^ future studies investigating its potential role in intestinal inflammation‐induced innate immune alterations would be warranted.

## 2 METHODS

### 2.1 Animal treatment

We used 5×FAD transgenic mice that overexpress the FAD mutant forms of human amyloid precursor protein (APP) containing the Swedish (K670N, M671L), the Florida (I716V), and the London (V717I) mutations along with presenilin 1 (PS1) (M146L, L286V) transgenes under the neuron‐specific mouse Thy‐1 promoter. Mice were purchased from Jackson Laboratory (Strain #006554). Seven‐week‐old mice were given autoclaved water or 3% DSS in autoclaved water (MP Biologicals) for 7 consecutive days, then switched to water. One day after treatment, mice were sacrificed for subsequent experiments.

### 2.2 Tissue processing

Mice were anesthetized using isoflurane, then transcardially perfused with 0.9% saline. Brains were then isolated and bisected into separate hemispheres. Both hemispheres were microdissected into the cortex/striatum, hippocampus, and the rest of the brain (midbrain and cerebellum). The left hemisphere was frozen on dry ice for biochemical studies while the right hemisphere was kept in 4% paraformaldehyde in cold 0.1 M phosphate buffer (pH 7.4) for three days, followed by incubation in 30% sucrose solution for immunohistochemistry. Brain tissue for immunohistochemistry was sectioned at a thickness of 40 µm on a dry ice‐cooled block on a sliding microtome (Leica, Wetzlar, Germany). The sections were stored at −20°C in a cryoprotective buffer consisting of 28% ethylene glycol, 23% glycol, and 0.05 M phosphate buffer until processing for immunohistochemistry.

### 2.3 16S rRNA sequencing

#### 2.3.1 Sequencing

Sequencing on the V3‐V4 fragment of the 16S rRNA, as well as sequencing using *Bifidobacterium* primers (Forward: CTCCTGGAAACGGGTGG; Reverse: GGTGTTCTTCCCGATATCTACA),^17^ were performed by RTL Genomics (Lubock, Texas) using DNA extracted from mouse stool samples on the Illumina MiSeq platform. After sequencing, the data were denoised and checked for chimeras, followed by quality checking. FASTA files are then generated, followed by operational taxonomic unit (out) selection, tree building, and taxonomic identification.

#### 2.3.2 Microbial community composition and differential abundance statistical analysis

Alpha‐diversity and richness using the ACE and Shannon indexes were calculated in R v4.0.3 using the *vegan*, *phyloseq*, and *microbiome* packages. Beta‐diversity was calculated in R as well, using the Bray–Curtis and Jaccard dissimilarity from OTU data. Statistical differences in alpha diversity and richness were performed using paired *t*‐tests. Differences among species, genus, family, order, class, and phylum levels were determined using analysis of composition of microbiomes with bias correction (ANCOMBC).

### 2.4 β‐Amyloid ELISAs

Cortex and hippocampal tissues were homogenized and sonicated in RIPA buffer (1 mL per 150 mg of tissue) and centrifuged at 45,000 × *g* for 30 min at 4°C. Supernatants were collected as the S1 fraction. The pellet was solubilized in 70% formic acid (FA; 2 µL per mg of tissue) and further centrifuged at 100,000 × *g* for 30 min at room temperature; the aqueous layer was collected as the formic acid fraction. Formic acid fractions were neutralized with 1 M Tris base in a 1:20 ratio prior to enzyme‐linked immunosorbent assays (ELISAs). Human β‐amyloid 40 and 42 concentrations were measured using Wako Human/Rat β Amyloid 40 (Code No. 294‐62501) and 42 (Code No. 290‐62601) ELISA kits as outlined in the manufacturer's protocol. Briefly, S1 and FA homogenates from either cortex and hippocampus, or serum were diluted in standard diluent (1:25 for cortex/hippocampus S1; 1:100,000 for cortex/hippocampus FA; 1:5 for plasma) to a total of 100 µL in duplicate and added to the antibody‐coated plates and incubated at 4°C overnight. The next day, plates were washed with wash solution (provided in kit), and 100 µL of horseradish peroxidase (HRP)‐conjugated antibody solution was added to wells and incubated at 4°C for 1 h. Plates were washed again, and 100 µL of 3,3′,5,5′‐tetramethylbenzidine (TMB) solution was added and incubated at room temperature for 30 min. Finally, 100 µL of stop solution was added to the wells in the plates to terminate the reaction, and the absorbance of each well was read at 450 nm for analysis.

**RESEARCH IN CONTEXT**

1. **Systematic review**: A comprehensive review of recent literature was conducted using PubMed. Evidence suggests that beta‐amyloid deposition may be influenced by alterations in the gut microbiome, as demonstrated in several mouse model studies, which could impact cognitive function. Specifically, peripheral inflammation associated with gut microbiota alterations is becoming recognized as a potential factor in Alzheimer's disease (AD) pathology. 
2. **Interpretation**: Acute colitis in 5xFAD mice shows significant changes in amyloid deposition, accompanied by increased peripheral immune cell infiltration, predominantly by monocytes, and increased efflux of beta‐amyloid into the bloodstream. These results suggest a clearance mechanism other than central nervous system (CNS) immune cells, highlighting the importance of the gut‐brain connection to AD. It also suggests that beta‐amyloid release into the bloodstream is in response to systemic inflammation, which could serve as an antimicrobial peptide to protect the brain.
3. **Future directions**: Identify the factors that affect peripheral immune cell involvement in CNS regulation and how these immune responses may shift with aging, impacting neuroinflammatory pathways. Determine the pathway by which beta‐amyloid is released into the bloodstream in response to intestinal inflammation.

### 2.5 Pro‐inflammatory cytokine measurements

RIPA fractions from cortex and hippocampus, or serum, were used to measure 10 cytokines: interferon‐λ, IL‐10, IL‐12p70, IL‐1β, IL‐2, IL‐4, IL‐5, IL‐6, keratinocyte‐derived chemokine (KC‐GRO), and TNFα. Cytokine measurement was performed using the Meso Scale Discovery (MSD, Rockville, MD) 96‐well Mouse Pro‐Inflammatory V‐PLEX Assay (Catalog #: K15048) as outlined in the manufacturer's protocol. Briefly, 25 µL of cortex and hippocampal lysate, or plasma mixed in a 1:1 ratio with the calibrator buffer in duplicate, were added to the antibody‐coated plate, which was then incubated for 2 h with vigorous shaking at room temperature. This was followed by washing with wash buffer (provided in the kit). After washing, 25 µL of the detection antibody was added and incubated for 2 h with vigorous shaking at room temperature. Lastly, the plate was washed with wash buffer before adding 150 µL 2 × MSD Read Buffer and immediately read on a Meso QuickPlex SQ 120.

### 2.6 Immunohistochemistry

Brain sections were permeabilized in phosphate buffered saline (PBS) containing 0.0025% Triton‐X100 and 5% donkey serum for 3 h. Then, sections were transferred to designated primary antibodies (intercellular adhesion molecule 1 [ICAM1], R & D Systems, catalog # AF796; HRP‐conjugated 4G8, Biolegend Catalog #800720; glial fibrillary acid protein [GFAP], abcam catalog # ab16997‐1; Iba1, Wako catalog # 019‐1974; LAMP1, abcam, catalog # ab25245, F4/80 (abcam, ab6640, 1:200), CD45 (Biolegend, 103102, 1:200) and Aβ42 (3D6, a gift from Lilly, 1:1000) in PBS containing Triton X‐100 and donkey serum overnight at room temperature. The next day, sections were washed three times with PBS for 10 min, followed by appropriate secondary antibody (Invitrogen) incubation for 2 h. Following secondary incubation, sections were washed three times and counterstained with Hoechst 33342 (1:10,000 in PBS) for 10 min and washed three times in PBS. Lastly, sections were mounted on charged slides and coverslipped using ProLong Gold anti‐fade reagent (Invitrogen).

For F4/80 immunohistochemistry, floating sections were placed in multi‐sample staining trays, and endogenous peroxidase activity was blocked (10% methanol, 3% H2O2 in PBS, 15 min). Tissue samples were permeabilized (1% Triton X‐100 in PBS, 30 min) and incubated overnight in primary antibody (1:1000). Sections were then rinsed in PBS then incubated in corresponding biotinylated secondary antibodies for 2 h. Following incubation, tissue sections were rinsed in PBS and incubated with Vectastain Elite ABC kit (Vector Laboratories, Burlingame, CA, USA) for enzyme conjugation. Finally, sections were developed using 0.05% diaminobenzidine, 0.5% nickel ammonium sulfate, and 0.03% hydrogen peroxide. Tissue sections were then mounted onto slides, dehydrated, and cover‐slipped.

### 2.7 Tissue imaging, quantification, and analysis

Tissue sections stained for Iba1, GFAP, and 4G8 were imaged on a Nikon A1R HD25 confocal microscope (Nikon, Tokyo, Japan) with the 10× objective. Tissue sections double labeled with Iba1 and 4G8 were imaged with the 40× objective. Iba1, GFAP, and 4G8 were quantified using ImageJ analysis software with four representative regions of interest per mouse to determine the percent positive area using consistent thresholds throughout the analysis (National Institutes of Health). To determine Iba1 and 4G8 colocalization, images were analyzed using ImageJ and the JACoP plugin to calculate the Mander's coefficient from four representative regions of interest per mouse using consistent thresholds throughout the analysis. The cortex and hippocampus were analyzed using the “Analyze Particles” method with ImageJ2 (Version 2.3.0) to quantify the percentile of the area of interest covered by 4G8, ICAM1, F4/80, Iba1, and GFAP. To determine statistical significance, *t*‐tests were performed using GraphPad Prism (Version 9.4.1).

### 2.8 Flow cytometry

To detect beta‐amyloid plaques in flow cytometry, 3 days prior to sacrifice, 5xFAD mice were injected intraperitoneally with methoxy‐X04 (10 mg/kg, Tocris). Thereafter, at sacrifice, single‐cell suspensions were prepared from mouse brain samples (around 400 mg) using the neural dissociation kit (Miltenyi catalog # 130‐092‐628). Samples were resuspended in 10 mL of PBS, followed by 5 mL Percoll (P1644) in PBS, and centrifugation (450 × *g*) at 4 degrees Celsius for 30 min with minimum acceleration and no brake. After centrifugation, the top layer of myelin was removed, and cells were resuspended and filtered through a 70 µm filter into a 50 mL tube, with a 10 mL PBS wash thereafter. Finally, 50 mL tubes were filled to the 45 mL mark with PBS and centrifuged (450 × *g*) at 4 degrees Celsius for 10 min. Excess PBS was removed, and the pelleted cells were resuspended in PBS for flow cytometry staining.

To stain cells for flow cytometry, first, they were blocked with anti‐mouse CD16/32 antibody (BD Biosciences, catalog # 553141) for 15 min in PBS. Afterward, cells were washed and centrifuged (1200 rpm) with PBS and placed in primary antibodies for 30 min, including LIVE/DEAD cell stain (L34967), fluorescein isothiocyanate (FITC) anti‐CD11c (BioLegend, catalog # 117305), PerCP‐Cy5.5 anti‐CD45 (BioLegend, catalog # 103131), APC anti‐CX3CR1 (BioLegend, catalog # 149007), AF700 anti‐LAMP1 (BioLegend, catalog # 121627), APC‐Cy7 anti‐CD11b (BD Pharmingen, catalog # 557657), PE anti‐P2RY12 (BioLegend, catalog # 848003), FITC anti‐CD45 (BioLegend, catalog # 103107), PerCP‐Cy5.5 anti‐CD3 (BioLegend, catalog # 100217), AF700 anti‐Ly6G (BioLegend, catalog # 127621), and PE‐Cy7 anti‐Ly6C (BioLegend, catalog # 128017). After primary antibody incubation, cells were washed again and fixed in 2% paraformaldehyde in PBS for 15 min unless performing intracellular staining. For intracellular staining, cells were washed and permeabilized with cell permeabilization buffer (Cell Signaling, catalog # 39487), then placed in primary antibody PE‐Cy7 anti‐CD68 (BioLegend, catalog # 565595) for 30 min. Once again, after 30 min, cells were washed with PBS, then fixed in 2% paraformaldehyde in PBS for 15 min.

### 2.9 Western blotting

Protein concentration was determined in cortical and hippocampal RIPA supernatants by Pierce bicinchoninic acid assay (BCA) assay (Thermo Fisher 23225). 20 µg of protein was loaded per sample into 4%–20% Bis‐Tris gels and separated by sodium dodecyl sulfate‐polyacrylamide gel electrophoresis (SDS‐PAGE), then transferred onto a polyvinylidene fluoride (PVDF) membrane. Protein levels were assessed by the addition of primary antibodies, followed by appropriate species‐specific secondary antibodies (LICOR IR dyes), then imaged using a LICOR machine. The following antibodies were used: ICAM1, R & D Systems, catalog # AF796; GFAP, abcam catalog # ab16997‐1; Iba1, Wako catalog # 019‐1974; LAMP1, abcam, catalog # ab25245; CD11b, Novus Biologicals, catalog # NB110‐89474; C‐term APP, Millipore catalog #171610; 6E10, Biolegend catalog # 803012; BACE1, Cell Signaling catalog # 5606; sAPPβ, IBL, catalog # 18957; CD10, Santa Cruz Biotechnology, catalog # sc‐46656; IDE, abcam catalog # ab32216; F4/80, abcam, catalog # ab6640.

### 2.10 PCR array for mouse tight junctions

Total RNA was extracted from the mouse hippocampus tissue using Trizol reagent. cDNA synthesis was carried out using Qiagen RT2 First strand Kit (Cat. No. 330404) with a genomic DNA elimination step using the total RNA of 500 ng‐1ug. Real‐time polymerase chain reaction (PCR) was carried out for tight junction gene expression using a PCR array from Qiagen RT^2^ Profiler  PCR Array Mouse Tight Junctions (PAMM‐143Z) by mixing the cDNA with SYBR Green Master Mix (RT^2^ SYBR Green qPCR‐ Cat. No. 330500) and dispensed into 96 well plate containing pre‐validated primers. Real‐time PCR was conducted in a Bio‐Rad – CFX96 thermal cycler with the recommended PCR conditions as per the PCR array kit guidelines. The analysis for the mouse tight junction gene expression was carried out using the Qiagen online analysis tool and represented the fold difference.

### 2.11 Human and animal rights

Animal procedures were performed in accordance with the recommendations of the National Research Council's “Guide for the Care and Use of Laboratory Animals” and were previously approved by the Massachusetts General Hospital's Institute of Animal Care and Use Committee (IACUC).

## 3 RESULTS

### 3.1 Acute colitis results in altered colonic structure and systemic inflammation

An increasing number of studies have connected gut health to AD neuropathology, prompting us to ask whether disruption of gut health through intestinal inflammation could impact AD pathology. We decided to use the 5xFAD mouse model, as this mouse exhibits rapid beta‐amyloid deposition as soon as eight weeks, high Aβ42 levels as early as 6 weeks, and aligns with the optimal age for successful and reproducible induction of acute colitis (using DSS) ranges between 6 and 8 weeks of age.^18^, ^19^ We only used female mice because they generally exhibit more profound pathology than male mice. To induce intestinal inflammation (colitis), 7‐week‐old 5xFAD mice were given ad libitum access to 3% DSS in autoclaved drinking water for 7 days, defined as an acute experimental colitis (acute colitis) paradigm, while control groups received autoclaved drinking water. For all experiments, on the seventh day, mice were sacrificed (8‐week‐old mice).

The most popular models of colitis include chemical induction (oxazolone, TNBS, DSS), adoptive T‐cell transfer in SCID or RAG2‐/‐ mice, IL‐10 knockout mice, and the SAMP1/YitFc mouse strain (spontaneous colitis model).^20^ We decided to use the DSS colitis model to avoid confounding effects of immunodeficiency on beta‐amyloid pathology (SCID/RAG2‐/‐) and because IL‐10 crossed with beta‐amyloid overexpressing mice have reduced deposition,^21^ showing that IL‐10 plays a role in beta‐amyloid pathology. DSS is thought to induce colitis by damaging the epithelial monolayer of the large intestine, which then allows proinflammatory intestinal contents such as bacteria or their byproducts to enter adjacent tissues. Thus, its effects on the gut are most similar to ulcerative colitis. Unlike human disease, T and B cells are not required for the development of colitis using DSS; hence, their contributions lie in research guided by understanding the contribution of the innate immune system to the development of intestinal inflammation.^22^ Nevertheless, the DSS model is highly translatable therapeutically, as many drug treatments in this model have been shown to aid in human autoimmune diseases.^23^

**FIGURE 1:**
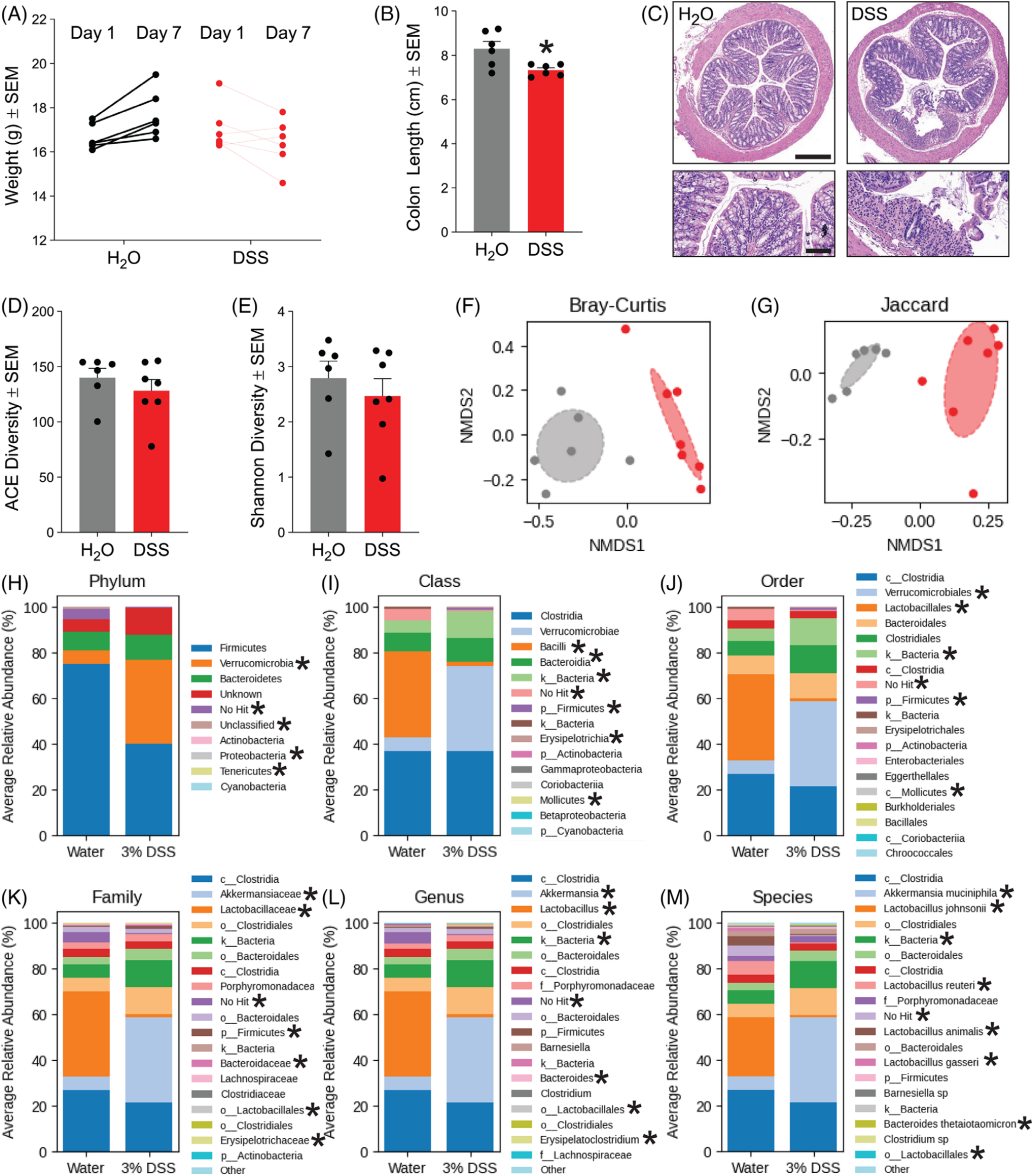
Acute colitis in 5xFAD mice causes colon crypt damage and promotes dysbiosis. (A) Mouse weights at the beginning and end of acute experimental colitis treatment (*n* = 6 mice/group). (B) Average mouse colon length (*n* = 6 mice/group, ^*^ *p* < 0.05). (C) Representative images of H & E‐stained mouse colon samples. Scale bars = 400 and 100 µm. (D) ACE alpha diversity calculation of water‐ and DSS‐treated 5xFAD mice (*n* = 6–7 mice/group, *p* < 0.05). (E) Shannon alpha diversity calculation of water‐ and DSS‐treated 5xFAD mice (*n* = 6‐7 mice/group, *p* < 0.05). (F) NMDS analysis of Bray–Curtis distance beta diversity in water‐ and DSS‐treated 5xFAD mice (*n* = 6–7 mice/group, *p* < 0.05). (G) NMDS analysis of Jaccard distance beta diversity in water‐ and DSS‐treated 5xFAD mice (*n* = 6–7 mice/group, *p* < 0.05). (H) Phylum, (I) class, (J) order, (K) family, (L) genus, and (M) species stacked bar plots from stool sample OTUs. Statistical analyses were carried out using two‐tailed unpaired *t*‐tests to compare two groups or using FDR‐corrected one‐way ANOVAs with Tukey's HSD post‐hoc for microbiome comparisons (*n* = 6–7 mice/group). ANOVA, analysis of variance; DSS, dextran sodium sulfate; FDR, false discovery rate; H & E, hematoxylin and eosin; HSD, honest significance test; NMDS, non‐metric multidimensional scaling; OTU, operational taxonomic unit.

We found that acute colitis in 5xFAD mice did not significantly alter weight (Figure 1A), though colon length was reduced (Figure 1B) and colonic architecture was drastically altered (Figure 1C) as evidenced by epithelial erosion and immune cell infiltration in hematoxylin and eosin (H & E) stained colon sections. Before sacrifice, we also collected stool samples for 16S sequencing to determine alterations in microbial diversity. While acute experimental colitis did not alter alpha diversity (species richness and evenness) as shown by the ACE (Figure 1D) and Shannon (Figure 1E) indices, non‐metric multidimensional scaling (NMDS) analysis using Bray–Curtis dissimilarity (Figure 1F) and Jaccard index (Figure 1G) showed dramatic alterations in the composition (beta diversity) of the microbiome. Verrucomicrobia, proteobacteria, and tenericutes phyla (Figure 1H) were the most affected by acute colitis, all experiencing increased colonization following acute colitis (Figure 1I; Figure S1F; Figure S2A; Table 1). Within classes, mollicutes and erysipelotrichia were elevated in stool samples, while bacilli were diminished (Figure 1J; Figure S1E; Figure S2B). At the order level, all the way to the species level, verrucomicrobiales colonization increased, with *Akkermansia muciniphila* being the prime species within this order, driving the changes. On the other hand, many *Lactobacillus* species were decreased, and this effect was observed all the way to the Lactobacillales order level (Figure 1J–M; Figure S1A–D; Figure S2C‐F). All significantly altered OTUs can be found in Table 1.

**TABLE 1:** OTU statistics.

OTU ID	Water average	DSS average	*p*‐value	Kingdom	Phylum	Class	Order	Family	Genus	Species
18	493.333	22	0.000	Bacteria	Firmicutes	Bacilli	Lactobacillales	Lactobacillaceae	Lactobacillus	Lactobacillus gasseri
19	12.5	1.571	0.000	Bacteria	Firmicutes	Clostridia	Unknown	Unknown	Unknown	Unknown
39	1503.167	99.429	0.000	Bacteria	Firmicutes	Clostridia	Unknown	Unknown	Unknown	Unknown
55	147	2.571	0.000	No Hit	No Hit	No Hit	No Hit	No Hit	No Hit	No Hit
79	1998.833	135	0.000	Bacteria	Firmicutes	Bacilli	Lactobacillales	Lactobacillaceae	Lactobacillus	Lactobacillus reuteri
85	199.167	0.571	0.000	Bacteria	Unclassified	Unclassified	Unclassified	Unclassified	Unclassified	Unclassified
96	244.333	6.429	0.000	Bacteria	Firmicutes	Clostridia	Clostridiales	Unknown	Unknown	Unknown
113	8952	404.286	0.000	Bacteria	Firmicutes	Bacilli	Lactobacillales	Lactobacillaceae	Lactobacillus	Lactobacillus johnsonii
121	526.667	35.857	0.000	Bacteria	Firmicutes	Clostridia	Unknown	Unknown	Unknown	Unknown
124	78.167	0.143	0.000	Bacteria	Firmicutes	Clostridia	Unknown	Unknown	Unknown	Unknown
125	1258.333	15	0.000	Bacteria	Firmicutes	Clostridia	Unknown	Unknown	Unknown	Unknown
134	42	1.714	0.000	Bacteria	Firmicutes	Clostridia	Clostridiales	Unknown	Unknown	Unknown
141	631.333	19.286	0.000	Bacteria	Unknown	Unknown	Unknown	Unknown	Unknown	Unknown
152	1107	29.571	0.000	No Hit	No Hit	No Hit	No Hit	No Hit	No Hit	No Hit
183	2340.667	515.857	0.000	Bacteria	Firmicutes	Clostridia	Unknown	Unknown	Unknown	Unknown
188	17	1.143	0.000	Bacteria	Firmicutes	Clostridia	Unclassified	Unclassified	Unclassified	Unclassified
12	115.333	2.429	0.001	Bacteria	Firmicutes	Clostridia	Unknown	Unknown	Unknown	Unknown
83	36.833	0.714	0.001	Bacteria	Firmicutes	Bacilli	Lactobacillales	Unknown	Unknown	Unknown
93	18.833	9.286	0.001	Bacteria	Firmicutes	Clostridia	Clostridiales	Unknown	Unknown	Unknown
150	2.5	906.286	0.001	Bacteria	Firmicutes	Clostridia	Unknown	Unknown	Unknown	Unknown
165	32.167	1.429	0.001	Bacteria	Firmicutes	Bacilli	Lactobacillales	Unknown	Unknown	Unknown
175	18.667	1254.429	0.001	Bacteria	Firmicutes	Clostridia	Unknown	Unknown	Unknown	Unknown
196	9	0.571	0.001	Bacteria	Actinobacteria	Unknown	Unknown	Unknown	Unknown	Unknown
20	1336	15,012.286	0.002	Bacteria	Verrucomicrobia	Verrucomicrobiae	Verrucomicrobiales	Akkermansiaceae	Akkermansia	Akkermansia muciniphila
76	67.667	24.571	0.003	Bacteria	Unknown	Unknown	Unknown	Unknown	Unknown	Unknown
120	233.833	25	0.003	Bacteria	Unknown	Unknown	Unknown	Unknown	Unknown	Unknown
193	15	2.571	0.003	Bacteria	Firmicutes	Clostridia	Clostridiales	Lachnospiraceae	Unclassified	Unclassified
64	548	24.429	0.004	Bacteria	Firmicutes	Clostridia	Unclassified	Unclassified	Unclassified	Unclassified
170	6.167	1	0.006	Bacteria	Actinobacteria	Unknown	Unknown	Unknown	Unknown	Unknown
11	127.5	13.857	0.008	Bacteria	Firmicutes	Clostridia	Unknown	Unknown	Unknown	Unknown
123	71	453.286	0.008	Bacteria	Verrucomicrobia	Verrucomicrobiae	Verrucomicrobiales	Akkermansiaceae	Akkermansia	Akkermansia muciniphila
177	1350	169.143	0.008	Bacteria	Firmicutes	Bacilli	Lactobacillales	Lactobacillaceae	Lactobacillus	Lactobacillus animalis
31	2.5	8.286	0.009	Bacteria	Proteobacteria	Betaproteobacteria	Burkholderiales	Unknown	Unknown	Unknown
159	12.5	6	0.009	Bacteria	Unknown	Unknown	Unknown	Unknown	Unknown	Unknown
60	7.167	0.286	0.011	No Hit	No Hit	No Hit	No Hit	No Hit	No Hit	No Hit
42	141.667	37.143	0.014	Bacteria	Firmicutes	Clostridia	Clostridiales	Unknown	Unknown	Unknown
122	6.667	77.714	0.014	Bacteria	Firmicutes	Clostridia	Unclassified	Unclassified	Unclassified	Unclassified
162	0.5	6	0.014	Bacteria	Firmicutes	Clostridia	Unclassified	Unclassified	Unclassified	Unclassified
116	1.833	7.286	0.015	Bacteria	Firmicutes	Clostridia	Clostridiales	Unknown	Unknown	Unknown
34	28.833	38.857	0.017	Bacteria	Firmicutes	Clostridia	Clostridiales	Clostridiaceae	Clostridium	Clostridium sp
48	2.833	0.143	0.017	Bacteria	Actinobacteria	Coriobacteriia	Eggerthellales	Eggerthellaceae	Adlercreutzia	Adlercreutzia equolifaciens
61	61	5.429	0.017	Bacteria	Firmicutes	Clostridia	Unclassified	Unclassified	Unclassified	Unclassified
107	101.667	3095.857	0.018	Bacteria	Firmicutes	Clostridia	Clostridiales	Unknown	Unknown	Unknown
154	5.167	350.571	0.020	Bacteria	Firmicutes	Clostridia	Unclassified	Unclassified	Unclassified	Unclassified
104	3	1.429	0.021	Bacteria	Firmicutes	Clostridia	Unknown	Unknown	Unknown	Unknown
197	0.167	19	0.022	Bacteria	Firmicutes	Clostridia	Unknown	Unknown	Unknown	Unknown
35	0.667	0.143	0.024	Bacteria	Actinobacteria	Unknown	Unknown	Unknown	Unknown	Unknown
105	1.333	18.143	0.026	No Hit	No Hit	No Hit	No Hit	No Hit	No Hit	No Hit
140	169.333	123.429	0.028	Bacteria	Firmicutes	Clostridia	Clostridiales	Unknown	Unknown	Unknown
184	2.667	1	0.028	Bacteria	Firmicutes	Clostridia	Unclassified	Unclassified	Unclassified	Unclassified
52	0.167	196.143	0.029	Bacteria	Firmicutes	Clostridia	Unclassified	Unclassified	Unclassified	Unclassified
24	1.167	16.286	0.033	Bacteria	Firmicutes	Clostridia	Unclassified	Unclassified	Unclassified	Unclassified
2	6	0.714	0.037	Bacteria	Firmicutes	Clostridia	Clostridiales	Ruminococcaceae	Anaerotruncus	Anaerotruncus colihominis
106	6.333	0.429	0.037	No Hit	No Hit	No Hit	No Hit	No Hit	No Hit	No Hit
190	8.5	0.143	0.039	Bacteria	Firmicutes	Clostridia	Clostridiales	Lachnospiraceae	Unknown	Unknown
4	1.667	293.286	0.042	Bacteria	Bacteroidetes	Bacteroidia	Bacteroidales	Bacteroidaceae	Bacteroides	Bacteroides thetaiotaomicron
8	84	92.286	0.042	Bacteria	Firmicutes	Clostridia	Unknown	Unknown	Unknown	Unknown
112	26.167	8.571	0.042	Bacteria	Firmicutes	Clostridia	Unclassified	Unclassified	Unclassified	Unclassified
51	8.5	11.429	0.048	Bacteria	Firmicutes	Clostridia	Clostridiales	Clostridiaceae	Clostridium	Clostridium sp
101	108.667	3297.429	0.050	Bacteria	Unknown	Unknown	Unknown	Unknown	Unknown	Unknown
100	1	0.571	0.051	Bacteria	Firmicutes	Clostridia	Unknown	Unknown	Unknown	Unknown
81	9	3.714	0.052	Bacteria	Actinobacteria	Coriobacteriia	Eggerthellales	Eggerthellaceae	Eggerthella	Eggerthella lenta
126	4.167	1.429	0.052	Bacteria	Firmicutes	Clostridia	Unknown	Unknown	Unknown	Unknown
46	8.667	3.429	0.056	Bacteria	Actinobacteria	Coriobacteriia	Eggerthellales	Eggerthellaceae	Enterorhabdus	Unknown
77	2.833	1.714	0.058	Bacteria	Firmicutes	Clostridia	Unclassified	Unclassified	Unclassified	Unclassified
87	0.167	6.143	0.058	Bacteria	Firmicutes	Clostridia	Unclassified	Unclassified	Unclassified	Unclassified
137	22.333	197.286	0.068	Bacteria	Unknown	Unknown	Unknown	Unknown	Unknown	Unknown
95	3.667	0.286	0.076	No Hit	No Hit	No Hit	No Hit	No Hit	No Hit	No Hit
192	6.167	0.429	0.076	Bacteria	Firmicutes	Clostridia	Unknown	Unknown	Unknown	Unknown
56	1.167	5.571	0.077	Bacteria	Verrucomicrobia	Verrucomicrobiae	Verrucomicrobiales	Akkermansiaceae	Akkermansia	Akkermansia muciniphila
167	1.667	1.571	0.077	Bacteria	Firmicutes	Unknown	Unknown	Unknown	Unknown	Unknown
63	5.833	6.286	0.087	Bacteria	Unknown	Unknown	Unknown	Unknown	Unknown	Unknown
128	7.5	55	0.087	Bacteria	Firmicutes	Clostridia	Unclassified	Unclassified	Unclassified	Unclassified
169	92	339.571	0.089	Bacteria	Firmicutes	Clostridia	Unknown	Unknown	Unknown	Unknown
54	0.167	16.143	0.092	Bacteria	Firmicutes	Clostridia	Clostridiales	Lachnospiraceae	Unknown	Unknown
69	3.833	0.714	0.097	No Hit	No Hit	No Hit	No Hit	No Hit	No Hit	No Hit
180	8.833	10.571	0.099	Bacteria	Firmicutes	Clostridia	Unclassified	Unclassified	Unclassified	Unclassified
186	78.333	729.429	0.101	Bacteria	Bacteroidetes	Bacteroidia	Bacteroidales	Porphyromonadacea	Unknown	Unknown
0	21.167	6.714	0.102	Bacteria	Bacteroidetes	Bacteroidia	Bacteroidales	Unknown	Unknown	Unknown
212	0	1.857	0.111	Bacteria	Firmicutes	Clostridia	Unclassified	Unclassified	Unclassified	Unclassified
204	14.667	11	0.123	Bacteria	Firmicutes	Clostridia	Unknown	Unknown	Unknown	Unknown
14	26.5	28.429	0.133	Bacteria	Firmicutes	Clostridia	Unclassified	Unclassified	Unclassified	Unclassified
92	0.833	1.286	0.135	Bacteria	Cyanobacteria	Unclassified	Chroococcales	Unclassified	Synechococcus	Synechococcus sp
47	34.167	11.286	0.145	Bacteria	Bacteroidetes	Bacteroidia	Bacteroidales	Unknown	Unknown	Unknown
211	0.333	30.714	0.146	Bacteria	Firmicutes	Clostridia	Unknown	Unknown	Unknown	Unknown
21	786	767.286	0.149	Bacteria	Firmicutes	Clostridia	Clostridiales	Unknown	Unknown	Unknown
62	1.667	50.143	0.149	Bacteria	Firmicutes	Clostridia	Unknown	Unknown	Unknown	Unknown
182	0.333	6.714	0.159	Bacteria	Firmicutes	Clostridia	Unknown	Unknown	Unknown	Unknown
99	3	2	0.164	Bacteria	Firmicutes	Clostridia	Unclassified	Unclassified	Unclassified	Unclassified
43	27.167	185.714	0.165	Bacteria	Firmicutes	Clostridia	Unknown	Unknown	Unknown	Unknown
164	4	34.143	0.185	Bacteria	Firmicutes	Clostridia	Unknown	Unknown	Unknown	Unknown
174	6	129.857	0.191	Bacteria	Firmicutes	Clostridia	Unclassified	Unclassified	Unclassified	Unclassified
206	53.667	58.143	0.197	Bacteria	Firmicutes	Clostridia	Unknown	Unknown	Unknown	Unknown
108	2	1.429	0.216	Bacteria	Actinobacteria	Coriobacteriia	Unknown	Unknown	Unknown	Unknown
130	21.667	406.571	0.218	Bacteria	Firmicutes	Clostridia	Clostridiales	Unknown	Unknown	Unknown
172	3.833	26.143	0.220	Bacteria	Firmicutes	Clostridia	Clostridiales	Unknown	Unknown	Unknown
40	46.833	146.714	0.228	Bacteria	Unknown	Unknown	Unknown	Unknown	Unknown	Unknown
28	298.5	726.286	0.238	Bacteria	Firmicutes	Clostridia	Unknown	Unknown	Unknown	Unknown
26	5.667	56.714	0.243	Bacteria	Firmicutes	Clostridia	Clostridiales	Unknown	Unknown	Unknown
171	374.167	43.857	0.244	Bacteria	Firmicutes	Clostridia	Unclassified	Unclassified	Unclassified	Unclassified
111	1.667	16.143	0.250	Bacteria	Firmicutes	Clostridia	Clostridiales	Unclassified	Unclassified	Unclassified
166	7.667	19	0.272	Bacteria	Firmicutes	Clostridia	Clostridiales	Unknown	Unknown	Unknown
143	0.667	19.571	0.283	Bacteria	Firmicutes	Clostridia	Unknown	Unknown	Unknown	Unknown
36	8.167	17.571	0.308	Bacteria	Firmicutes	Clostridia	Clostridiales	Lachnospiraceae	Coprococcus	Unknown
109	1.667	4.429	0.328	Bacteria	Unclassified	Unclassified	Unclassified	Unclassified	Unclassified	Unclassified
155	6.167	7.429	0.329	Bacteria	Firmicutes	Clostridia	Clostridiales	Unknown	Unknown	Unknown
129	16	107.143	0.348	Bacteria	Bacteroidetes	Bacteroidia	Bacteroidales	Porphyromonadacea	Unknown	Unknown
66	102.333	395.714	0.355	Bacteria	Firmicutes	Clostridia	Unknown	Unknown	Unknown	Unknown
15	187.167	84.429	0.359	No Hit	No Hit	No Hit	No Hit	No Hit	No Hit	No Hit
103	2.333	27.571	0.385	Bacteria	Firmicutes	Clostridia	Unknown	Unknown	Unknown	Unknown
176	2.333	16.143	0.398	Bacteria	Unknown	Unknown	Unknown	Unknown	Unknown	Unknown
88	0.833	6.429	0.423	Bacteria	Unknown	Unknown	Unknown	Unknown	Unknown	Unknown
185	1.833	10.143	0.438	Bacteria	Unclassified	Unclassified	Unclassified	Unclassified	Unclassified	Unclassified
58	15.167	68.286	0.452	Bacteria	Firmicutes	Erysipelotrichia	Erysipelotrichales	Erysipelotrichaceae	Erysipelatoclostr	Clostridium cocleatum
205	6.333	228.286	0.469	Bacteria	Firmicutes	Clostridia	Unknown	Unknown	Unknown	Unknown
132	1.667	5.143	0.501	Bacteria	Firmicutes	Erysipelotrichia	Erysipelotrichales	Unknown	Unknown	Unknown
179	1.333	143.143	0.502	Bacteria	Firmicutes	Clostridia	Unclassified	Unclassified	Unclassified	Unclassified
187	1.5	4.857	0.529	Bacteria	Firmicutes	Clostridia	Unknown	Unknown	Unknown	Unknown
89	728.5	1859	0.541	Bacteria	Bacteroidetes	Bacteroidia	Bacteroidales	Unknown	Unknown	Unknown
37	1.667	1.571	0.557	Bacteria	Firmicutes	Clostridia	Clostridiales	Catabacteriaceae	Catabacter	Catabacter hongkongensis
139	0.5	3.143	0.573	Bacteria	Unknown	Unknown	Unknown	Unknown	Unknown	Unknown
13	90.333	426.143	0.586	Bacteria	Firmicutes	Unknown	Unknown	Unknown	Unknown	Unknown
173	12	6.143	0.596	Bacteria	Firmicutes	Clostridia	Unclassified	Unclassified	Unclassified	Unclassified
86	1.667	4	0.611	Bacteria	Firmicutes	Clostridia	Unclassified	Unclassified	Unclassified	Unclassified
22	2.333	11.714	0.622	Bacteria	Firmicutes	Clostridia	Clostridiales	Ruminococcaceae	Ruminococcus	Unknown
198	2.333	12.429	0.656	Bacteria	Firmicutes	Clostridia	Unknown	Unknown	Unknown	Unknown
25	0.667	39.857	0.659	Bacteria	Firmicutes	Clostridia	Unknown	Unknown	Unknown	Unknown
144	1.667	2.143	0.672	Bacteria	Firmicutes	Clostridia	Unclassified	Unclassified	Unclassified	Unclassified
73	446.5	1038.429	0.694	Bacteria	Unknown	Unknown	Unknown	Unknown	Unknown	Unknown
72	28.833	14.429	0.696	Bacteria	Actinobacteria	Unknown	Unknown	Unknown	Unknown	Unknown
157	52.333	111.143	0.726	Bacteria	Firmicutes	Clostridia	Unknown	Unknown	Unknown	Unknown
161	3	5.857	0.739	Bacteria	Firmicutes	Clostridia	Clostridiales	Clostridiaceae	Clostridium	Clostridium sp
78	18.667	61.571	0.759	Bacteria	Firmicutes	Clostridia	Unknown	Unknown	Unknown	Unknown
82	38	120.571	0.768	Bacteria	Firmicutes	Clostridia	Clostridiales	Unknown	Unknown	Unknown
208	159	565	0.774	Bacteria	Firmicutes	Clostridia	Unknown	Unknown	Unknown	Unknown
118	70.333	154.857	0.780	Bacteria	Firmicutes	Clostridia	Unknown	Unknown	Unknown	Unknown
138	584.5	935.286	0.802	Bacteria	Bacteroidetes	Bacteroidia	Bacteroidales	Unclassified	Unclassified	Unclassified
30	34.167	125	0.816	Bacteria	Bacteroidetes	Bacteroidia	Bacteroidales	Unknown	Unknown	Unknown
153	6	23.714	0.833	Bacteria	Firmicutes	Clostridia	Clostridiales	Unknown	Unknown	Unknown
181	0.167	2.286	0.868	Bacteria	Firmicutes	Clostridia	Clostridiales	Clostridiaceae	Clostridium	Clostridium sp
44	92.5	491.857	0.882	Bacteria	Firmicutes	Clostridia	Unknown	Unknown	Unknown	Unknown
210	166.833	125.571	0.883	Bacteria	Firmicutes	Clostridia	Unknown	Unknown	Unknown	Unknown
209	79.833	126.143	0.890	Bacteria	Bacteroidetes	Bacteroidia	Bacteroidales	Unknown	Unknown	Unknown
201	111	154.286	0.896	Bacteria	Bacteroidetes	Bacteroidia	Bacteroidales	Porphyromonadacea	Barnesiella	Barnesiella sp
50	317.333	1790.571	0.910	Bacteria	Firmicutes	Clostridia	Unknown	Unknown	Unknown	Unknown
97	2.167	3.143	0.913	Bacteria	Firmicutes	Bacilli	Bacillales	Unclassified	Dehalobacterium	Dehalobacterium sp
80	304.167	827.143	0.939	Bacteria	Firmicutes	Clostridia	Unknown	Unknown	Unknown	Unknown
9	1.5	29.714	0.943	Bacteria	Firmicutes	Clostridia	Clostridiales	Unknown	Unknown	Unknown
23	10	37.143	0.945	Bacteria	Firmicutes	Clostridia	Clostridiales	Lachnospiraceae	Unclassified	Unclassified
168	38.167	54.714	0.950	Bacteria	Unknown	Unknown	Unknown	Unknown	Unknown	Unknown
110	551.167	420.857	0.954	Bacteria	Bacteroidetes	Bacteroidia	Bacteroidales	Porphyromonadacea	Unknown	Unknown
142	3.5	18.429	0.958	Bacteria	Bacteroidetes	Bacteroidia	Bacteroidales	Unknown	Unknown	Unknown
98	2.833	70.857	0.966	Bacteria	Firmicutes	Clostridia	Clostridiales	Unclassified	Unclassified	Unclassified
71	6	6.857	0.992	Bacteria	Firmicutes	Clostridia	Clostridiales	Lachnospiraceae	Unclassified	Unclassified
1	96.833	0	1.000	Bacteria	Firmicutes	Clostridia	Unknown	Unknown	Unknown	Unknown
3	0	1.571	1.000	Bacteria	Unclassified	Unclassified	Unclassified	Unclassified	Unclassified	Unclassified
6	31.833	0	1.000	Bacteria	Bacteroidetes	Bacteroidia	Bacteroidales	Unknown	Unknown	Unknown
10	6.5	0	1.000	Bacteria	Firmicutes	Clostridia	Clostridiales	Unclassified	Unclassified	Unclassified
16	74.167	0	1.000	Bacteria	Firmicutes	Clostridia	Unknown	Unknown	Unknown	Unknown
17	0	13.857	1.000	Bacteria	Firmicutes	Clostridia	Unclassified	Unclassified	Unclassified	Unclassified
27	0	5.286	1.000	Bacteria	Firmicutes	Unknown	Unknown	Unknown	Unknown	Unknown
32	0	34.286	1.000	Bacteria	Firmicutes	Clostridia	Unclassified	Unclassified	Unclassified	Unclassified
33	0	8.714	1.000	Bacteria	Firmicutes	Clostridia	Clostridiales	Unknown	Unknown	Unknown
38	142.333	0	1.000	Bacteria	Firmicutes	Clostridia	Clostridiales	Unknown	Unknown	Unknown
45	81.5	0	1.000	Bacteria	Firmicutes	Clostridia	Unknown	Unknown	Unknown	Unknown
49	0	6.143	1.000	Bacteria	Firmicutes	Clostridia	Clostridiales	Ruminococcaceae	Anaerotruncus	Anaerotruncus colihominis
53	0	136.571	1.000	Bacteria	Firmicutes	Clostridia	Unclassified	Unclassified	Unclassified	Unclassified
57	0	11.857	1.000	Bacteria	Firmicutes	Clostridia	Clostridiales	Unknown	Unknown	Unknown
59	7.333	0	1.000	Bacteria	Firmicutes	Clostridia	Clostridiales	Unknown	Unknown	Unknown
65	0	45	1.000	Bacteria	Proteobacteria	Gammaproteobacter	Enterobacteriales	Enterobacteriaceae	Escherichia	Escherichia coli
68	0	1.143	1.000	Bacteria	Firmicutes	Clostridia	Unknown	Unknown	Unknown	Unknown
70	0	16.714	1.000	Bacteria	Firmicutes	Clostridia	Clostridiales	Unknown	Unknown	Unknown
74	104.5	0	1.000	Bacteria	Firmicutes	Clostridia	Clostridiales	Unknown	Unknown	Unknown
75	0	41	1.000	Bacteria	Firmicutes	Unknown	Unknown	Unknown	Unknown	Unknown
84	0	8.857	1.000	Bacteria	Firmicutes	Clostridia	Clostridiales	Unclassified	Unclassified	Unclassified
90	0	3.143	1.000	Bacteria	Firmicutes	Clostridia	Clostridiales	Unknown	Unknown	Unknown
91	0	1.429	1.000	Bacteria	Firmicutes	Clostridia	Clostridiales	Unknown	Unknown	Unknown
94	0	18.286	1.000	Bacteria	Firmicutes	Clostridia	Clostridiales	Unknown	Unknown	Unknown
115	0	13	1.000	Bacteria	Firmicutes	Clostridia	Clostridiales	Unknown	Unknown	Unknown
117	0	13.143	1.000	Bacteria	Firmicutes	Clostridia	Unknown	Unknown	Unknown	Unknown
119	10.333	0	1.000	Bacteria	Firmicutes	Clostridia	Unclassified	Unclassified	Unclassified	Unclassified
127	0	2	1.000	Bacteria	Firmicutes	Clostridia	Clostridiales	Unclassified	Unclassified	Unclassified
131	0	1.714	1.000	Bacteria	Firmicutes	Clostridia	Clostridiales	Unclassified	Unknown	Unknown
133	28.333	0	1.000	Bacteria	Unknown	Unknown	Unknown	Unknown	Unknown	Unknown
145	0	22.286	1.000	Bacteria	Firmicutes	Clostridia	Unclassified	Unclassified	Unclassified	Unclassified
146	0	1.714	1.000	Bacteria	Firmicutes	Clostridia	Unknown	Unknown	Unknown	Unknown
147	8	0	1.000	Bacteria	Firmicutes	Clostridia	Unknown	Unknown	Unknown	Unknown
148	0	16.571	1.000	Bacteria	Firmicutes	Clostridia	Clostridiales	Unknown	Unknown	Unknown
149	10.333	0	1.000	No Hit	No Hit	No Hit	No Hit	No Hit	No Hit	No Hit
151	30.833	0	1.000	Bacteria	Firmicutes	Clostridia	Unknown	Unknown	Unknown	Unknown
156	31.167	0	1.000	Bacteria	Firmicutes	Clostridia	Unknown	Unknown	Unknown	Unknown
158	0	1	1.000	Bacteria	Verrucomicrobia	Verrucomicrobiae	Verrucomicrobiales	Akkermansiaceae	Akkermansia	Akkermansia muciniphila
160	1	0	1.000	Bacteria	Firmicutes	Clostridia	Unknown	Unknown	Unknown	Unknown
178	0	10	1.000	Bacteria	Firmicutes	Unknown	Unknown	Unknown	Unknown	Unknown
191	0	2.143	1.000	Bacteria	Firmicutes	Clostridia	Clostridiales	Unclassified	Unclassified	Unclassified
202	20.333	0	1.000	Bacteria	Firmicutes	Clostridia	Clostridiales	Eubacteriaceae	Eubacterium	Eubacterium oxidoreducens
203	66	0	1.000	Bacteria	Firmicutes	Clostridia	Unknown	Unknown	Unknown	Unknown
207	5.167	0	1.000	Bacteria	Firmicutes	Clostridia	Clostridiales	Ruminococcaceae	Anaerotruncus	Anaerotruncus colihominis
213	0.833	31.571	1.000	Bacteria	Tenericutes	Mollicutes	Unclassified	Unclassified	Unclassified	Unclassified
214	0	6.857	1.000	Bacteria	Firmicutes	Clostridia	Unclassified	Unclassified	Unclassified	Unclassified
5	0	0.571		Bacteria	Firmicutes	Erysipelotrichia	Erysipelotrichales	Unknown	Unknown	Unknown
7	0	1.857		Bacteria	Unknown	Unknown	Unknown	Unknown	Unknown	Unknown
29	0.167	0.571		Bacteria	Firmicutes	Clostridia	Unclassified	Unclassified	Unclassified	Unclassified
41	0	7.143		Bacteria	Firmicutes	Clostridia	Unclassified	Unclassified	Unclassified	Unclassified
67	24.333	0		Bacteria	Firmicutes	Clostridia	Unclassified	Unclassified	Unclassified	Unclassified
102	0	0.571		Bacteria	Firmicutes	Clostridia	Clostridiales	Unknown	Unknown	Unknown
114	0	2.571		Bacteria	Firmicutes	Clostridia	Unclassified	Unclassified	Unclassified	Unclassified
135	0	0.429		Bacteria	Firmicutes	Clostridia	Clostridiales	Unknown	Unknown	Unknown
136	0	0.429		Bacteria	Tenericutes	Mollicutes	Unclassified	Unclassified	Unclassified	Unclassified
163	0	1.571		No Hit	No Hit	No Hit	No Hit	No Hit	No Hit	No Hit
189	0.167	0.857		No Hit	No Hit	No Hit	No Hit	No Hit	No Hit	No Hit
194	0	15.286		Bacteria	Firmicutes	Clostridia	Unclassified	Unclassified	Unclassified	Unclassified
195	0	5		Bacteria	Firmicutes	Clostridia	Unclassified	Unclassified	Unclassified	Unclassified
199	0	1.571		Bacteria	Firmicutes	Clostridia	Unclassified	Unclassified	Unclassified	Unclassified
200	0.833	0		Bacteria	Unknown	Unknown	Unknown	Unknown	Unknown	Unknown
215	2.333	0		No Hit	No Hit	No Hit	No Hit	No Hit	No Hit	No Hit
216	0.333	1		Bacteria	Unknown	Unknown	Unknown	Unknown	Unknown	Unknown

Abbreviations: DSS, dextran sodium sulfate; OUT, operational taxonomic units.

**FIGURE 2:**
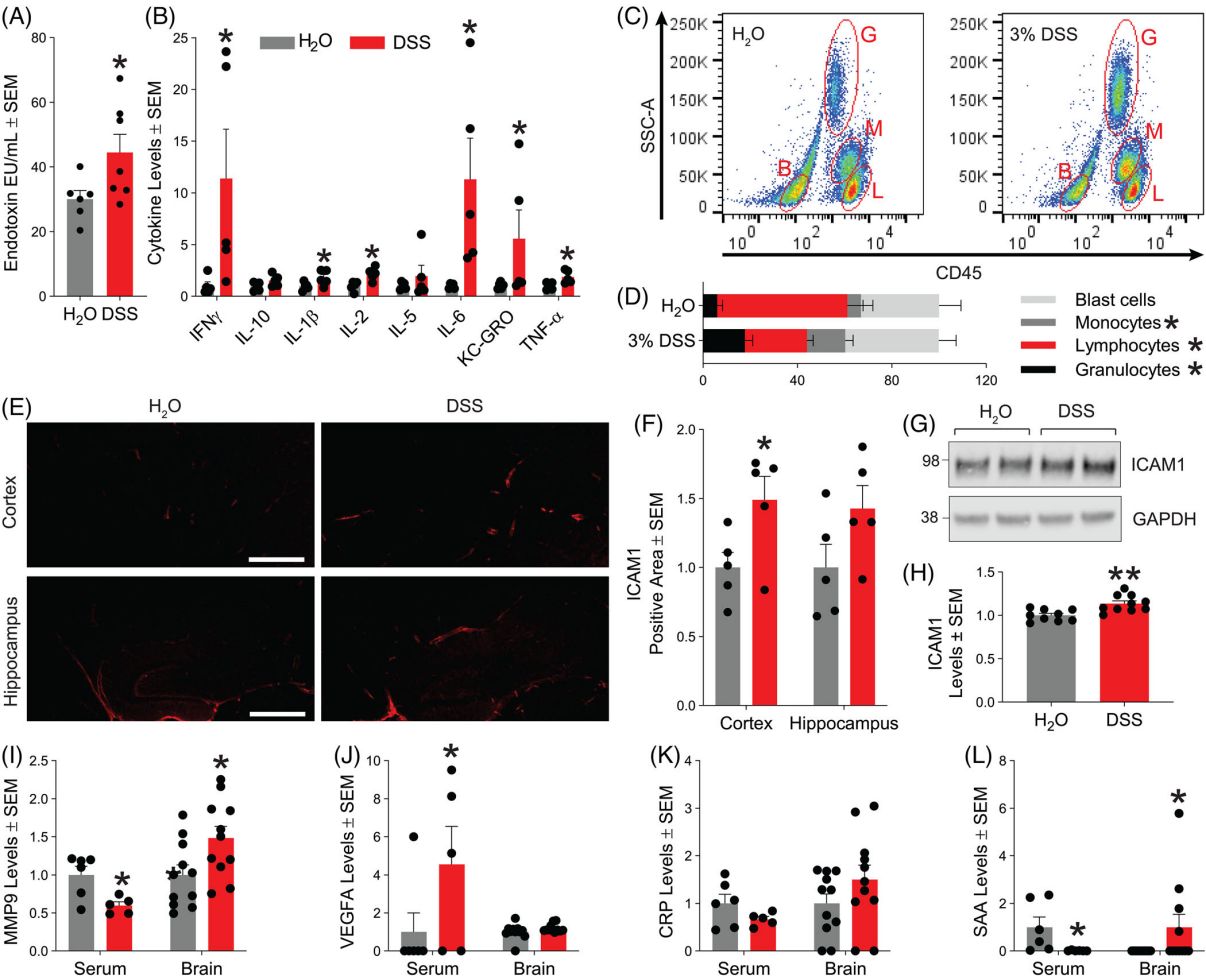
Acute colitis in 5xFAD mice induces systemic inflammation. (A) Mouse serum endotoxin levels (*n* = 6–7 mice/group, *p < 0.05). (B) Mouse serum pro‐inflammatory cytokine levels (*n* = 6 mice/group, *p < 0.05). (C) Scatter plots from mouse whole blood samples stained for CD45 to identify white blood cell populations (G = granulocytes; M = monocytes; L = lymphocytes; B = blast cells). (D) Stacked bar plot showing relative population of white blood cells in mouse whole blood (*n* = 6 mice/group, *p < 0.05). (E) Representative images of the mouse brain cortex and hippocampus stained for ICAM1. (F) Quantification of percent positive area covered by ICAM1 immunoreactivity in the cortex and hippocampus from mouse brain sections (*n* = 5 mice/group, *p < 0.05). (G) Western blot from mouse brain cortical homogenates probed for ICAM1 and GAPDH. (H) Quantification of ICAM1 levels normalized to GAPDH in mouse brain cortical homogenates (*n* = 9–10 mice/group from two independent experiments, **p < 0.01). (I) MMP9 levels in serum (*n* = 5‐6 mice/group, *p < 0.05) and in mouse brain cortical homogenates (*n* = 11/group, *p < 0.05). (J) VEGFA levels in serum (*n* = 5–6 mice/group, *p < 0.05) and in mouse brain cortical homogenates (*n* = 11/group, *p < 0.05). (K) CRP levels in serum (*n* = 5–6 mice/group, *p < 0.05) and in mouse brain cortical homogenates (*n* = 11/group, *p < 0.05). (L) SAA levels in serum (*n* = 5–6 mice/group, *p < 0.05) and in mouse brain cortical homogenates (*n* = 11/group, *p < 0.05). Statistical analyses were performed by two‐tailed unpaired *t*‐tests. CRP, C‐reactive protein; GAPDH, glyceraldehyde‐3‐phosphate dehydrogenase; ICAM1, intercellular adhesion molecule 1; MMP9, matrix metalloproteinase 9; SAA, serum amyloid A.

To examine the effects of intestinal inflammation on systemic inflammation, we measured endotoxin levels in serum samples and observed markedly elevated levels (Figure 2A) along with increased concentrations of several pro‐inflammatory cytokines (Figure 2B), including interferon γ (IFNγ), IL‐1β, IL‐2, IL‐5, IL‐6, KC‐GRO, and TNF‐α, in mouse serum samples from DSS‐treated 5xFAD mice compared to controls. Increased percentages of neutrophils, as well as monocytes, and diminished lymphocytes were also observed in whole blood samples by flow cytometry (Figure 2C–D), consistent with published data on DSS‐induced colitis. We also observed the presence of blood by Hemoccult in the stool of mice receiving DSS (data not shown). Overall, acute colitis in 5xFAD mice was successfully induced, with several hallmarks of intestinal and systemic inflammation observed in our model.

Next, we tested whether the robust systemic inflammation induced by acute colitis could affect neuroinflammation. Through brain tissue immunofluorescence, we quantified changes in ICAM1 fluorescence as measures of brain endothelial activation in the mouse brain cortex and hippocampus (Figure 2E). ICAM1 expression in the cortex (Figure 2F) was significantly increased in response to acute colitis, while a trend toward increased expression was observed in the hippocampus. Western blot analysis of ICAM1 expression revealed similar findings (Figure 2G–H). The endothelial activation was accompanied by increased brain matrix metalloproteinase‐9 (MMP9), C‐reactive protein (CRP), and serum amyloid A (SAA), along with a nonsignificant increase in vascular endothelial growth factor A (VEGFA) (Figure 2I–L). Interestingly, in serum, while VEGFA levels were increased by acute colitis (Figure 2J), MMP9 and SAA levels were decreased, and CRP remained unchanged. We also tested whether endothelial activation suggested BBB breakdown. Interestingly, we found decreases in RNA transcripts for several tight junction proteins, including ZO3 (*Tjp3*), occludin (*Ocln*), Par‐3 (*Pard3*), Catenin‐α3 (*Ctnna3*), and claudin 12 (*Cldn12*) (Figure S3A, B).

**FIGURE 3:**
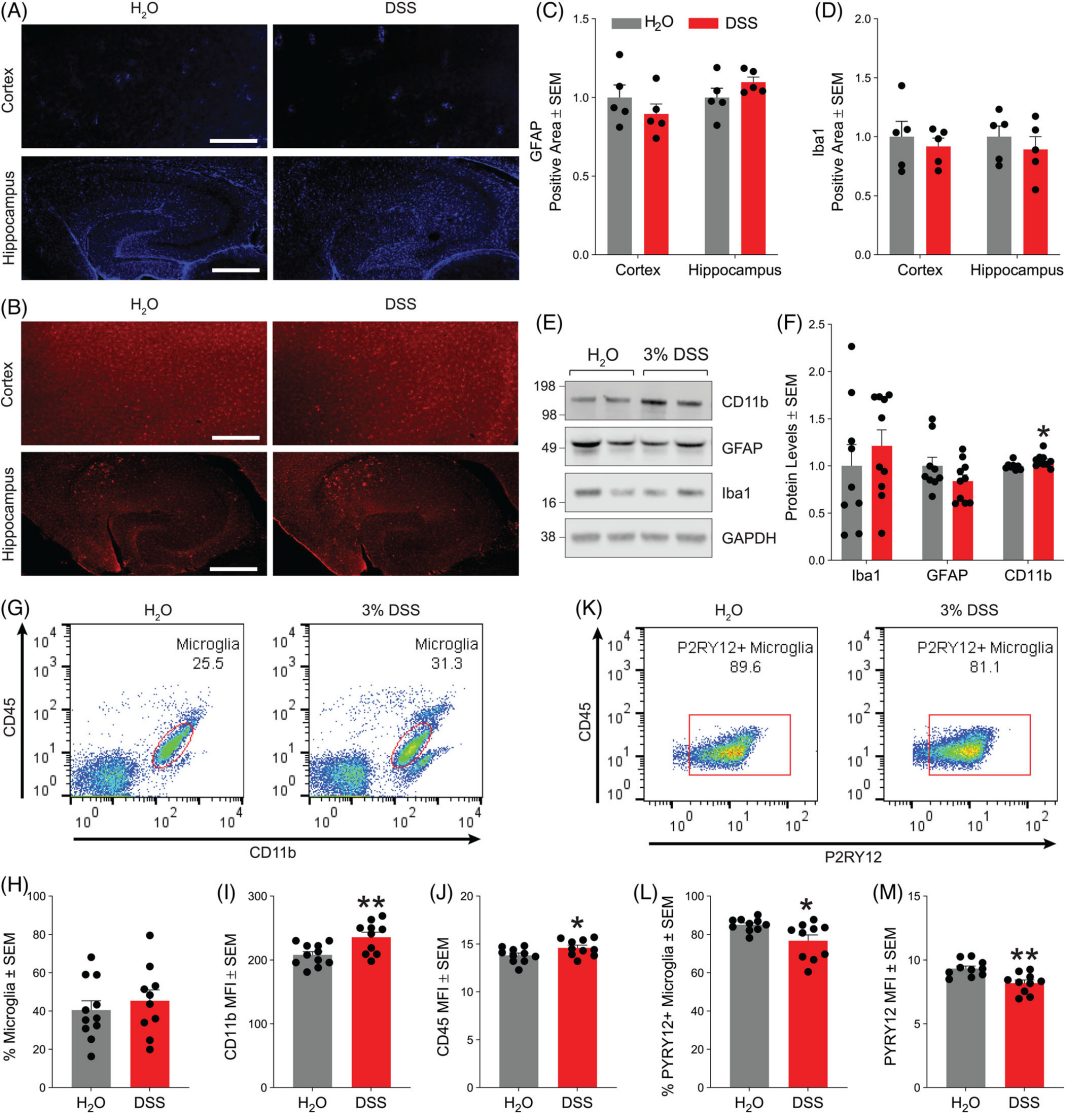
Neuroinflammation in the 5xFAD brain in response to acute colitis. Representative images of the mouse brain cortex and hippocampus fluorescently stained for (A) GFAP and (B) Iba1. Scale bar = 300 µm. (C) Normalized percent positive area occupied by GFAP immunoreactivity in the mouse brain cortex and hippocampus (*n* = 5 mice/group, *p < 0.05). (D) Normalized percent positive area occupied by Iba1 immunoreactivity in the mouse brain cortex and hippocampus (*n* = 5 mice/group, *p < 0.05). (E) Representative Western blots from mouse cortical homogenates probed for CD11b, GFAP, Iba1, and GAPDH. (F) Quantification of CD11b, GFAP, and Iba1 markers normalized to GAPDH (*n* = 9–10 mice/group from two independent experiments, *p < 0.05). (G) Flow cytometry scatter plots of mouse brain single cell suspensions stained for CD45 and CD11b to identify microglia (CD11b+CD45low). (H) Percentage of the microglial population defined by CD11b+CD45low in mouse brain single cell suspensions. (I) CD11b and (J) CD45 mean fluorescence intensity within the CD11b+CD45low gated population of microglia. (K) Flow cytometry scatter plots of gated‐microglia (CD11b+CD45low) positive for P2RY12 immunoreactivity from mouse brain single cell suspensions. (L) Percentage of the microglial population (CD11b+CD45low) that is positive for P2RY12 immunoreactivity. (M) P2RY12 mean fluorescence intensity within the CD11b+CD45low gated population of microglia (*n* = 10–11 from two independent experiments: *p < 0.05, **p < 0.01). Statistical analyses were performed by two‐tailed unpaired *t*‐tests. GAPDH, glyceraldehyde‐3‐phosphate dehydrogenase; GFAP, glial fibrillary acid protein.

### 3.2 Neuroinflammation due to acute experimental colitis in 5xFAD mice

Neuroinflammation in the brain in response to acute colitis was further explored by assessing astrogliosis and microgliosis based on GFAP (Figure 3A) and Iba1 (Figure 3B) immunofluorescence in brain tissue sections and by Western blotting. While we did not detect alterations in microglial or astrocytic burden in either the cortex or hippocampus (Figure 3C–D; Figure 3E–F), Western blotting in brain homogenates from two independent studies showed an increase in CD11b levels following DSS treatment, indicating microglial activation^24^, ^25^ (Figure 3E–F). Flow cytometry was performed on brain single cell suspensions to further characterize activated microglia during acute colitis using several markers, including CD45, CD11b, P2RY12, CD11c, CD68, CX3CR1, and LAMP1. After gating flow cytometry events to identify single live cells, we used CD11b and CD45 fluorescence to identify the microglia population (Figure S4). Then, within the microglial population, we measured the microglia marker fluorescence and percent microglia population positive for these markers. The percentage of microglia within the brain single cell suspensions was not altered by acute colitis (Figure 3G–H). Both CD11b (Figure 3I), similar to Western blot findings, and CD45 (Figure 3J) fluorescence was markedly increased while a signature marker of homeostatic microglia, P2RY12^26^, ^27^, ^28^, ^29^ (Figure 3K–L), was significantly decreased. Further, the percent positive population for P2RY12 (Figure 3L) was significantly reduced as well, suggesting that acute colitis activated microglia toward a dystrophic state.^30^ Meanwhile, CD11c, CD68, LAMP1, and CX3CR1 microglial markers remained unchanged (Figure S5A–F).

Given the robust degree of systemic inflammation, we next explored the effects of acute colitis on the concentration of pro‐inflammatory cytokines in the brain. For this purpose, we performed MSD assays on cortical and hippocampal homogenates from two independent studies and found regional differences in cytokine production in the brain. While IL‐4 levels were elevated in cortical homogenates from DSS‐treated mice, IL‐2 levels were substantially decreased (Figure S6A). We also observed nonsignificant trends toward an increase in IFN‐γ and KC‐GRO in cortical homogenates from acute colitis mice, while all other cytokines remained unchanged. In contrast, hippocampal homogenates revealed increased IL‐2 and IL‐6 cytokine levels following acute colitis (Figure S6B). Hippocampal IL‐4 levels trended upward due to acute colitis, but they did not reach statistical significance.

### 3.3 Acute colitis in 5xFAD reduces beta‐amyloid deposition in the brain

**FIGURE 4:**
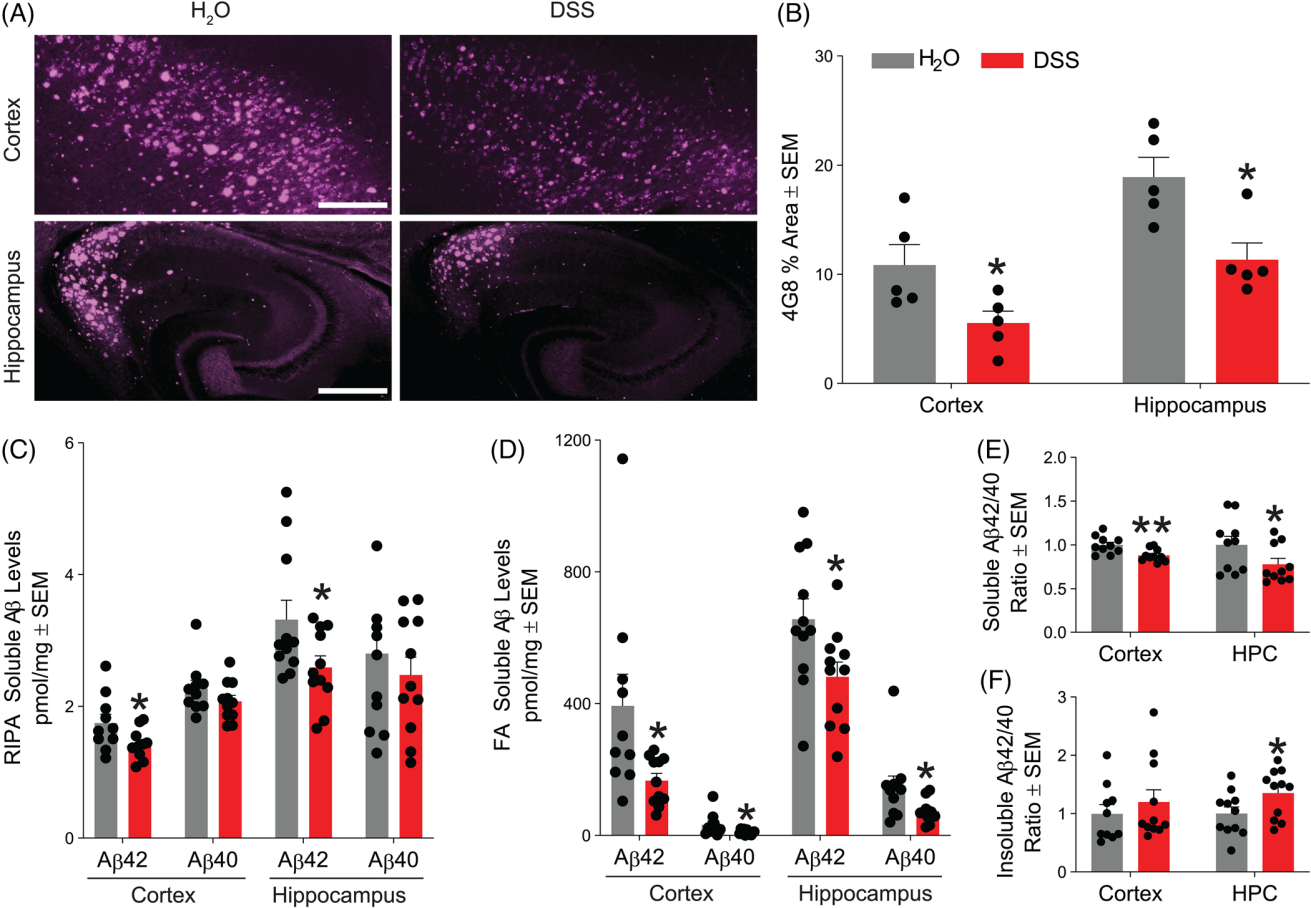
Reduced Aβ deposition and insoluble accumulation following acute colitis in 5xFAD mice. (A) Representative images of mouse cortex and hippocampus stained for the 4G8 marker to detect Aβ. Scale bar = 300 µm. (B) Quantification of percent positive area covered by 4G8 in the cortex and hippocampus (*n* = 5 mice/group, *p < 0.05). (C) RIPA soluble Aβ42 and Aβ40 levels in the mouse cortex and hippocampus (*n* = 10–11 mice/group from two independent experiments, *p < 0.05). (D) FA soluble Aβ42 and Aβ40 levels in the mouse cortex and hippocampus (*n* = 10–11 mice/group from two independent experiments, *p < 0.05). (E) Average normalized RIPA soluble Aβ42/40 ratios in mouse cortical and HPC homogenates (*n* = 9–10 mice/group, *p < 0.05, **p < 0.01). (F) Average normalized FA soluble Aβ42/40 ratios in mouse cortical and HPC homogenates (*n* = 9–10 mice/group, *p < 0.05, **p < 0.01). Statistical analyses were performed by two‐tailed unpaired *t*‐tests. FA, formic acid; HPC, hippocampal.

Next, we performed immunofluorescence on brain tissue sections for beta‐amyloid using the antibody 4G8 (Figure 4A) and quantified the percent positive area in the cortex and hippocampus. Surprisingly, we observed that acute colitis led to a pronounced reduction in beta‐amyloid deposition in both the cortex and hippocampus (Figure 4B). These results were corroborated by ELISAs targeting Aβ42 and Aβ40 on cortical and hippocampal homogenates from two independent studies, revealing a significant decrease in soluble Aβ42 in both regions following acute colitis. A nonsignificant downward trend was observed for Aβ40 (Figure 4C). Additionally, acute colitis significantly reduced insoluble (formic‐acid soluble) Aβ42 and Aβ40 in both the cortex and hippocampus (Figure 4D), mirroring the reduction in beta‐amyloid deposition observed with 4G8 immunofluorescence. The soluble Aβ 42/40 ratio was decreased (Figure 4E) while the insoluble ratio was increased (Figure 4F) due to the acute colitis treatment.

### 3.4 Acute colitis in 5xFAD mice increases leukocyte infiltration into the brain and beta‐amyloid efflux into the bloodstream

**FIGURE 5:**
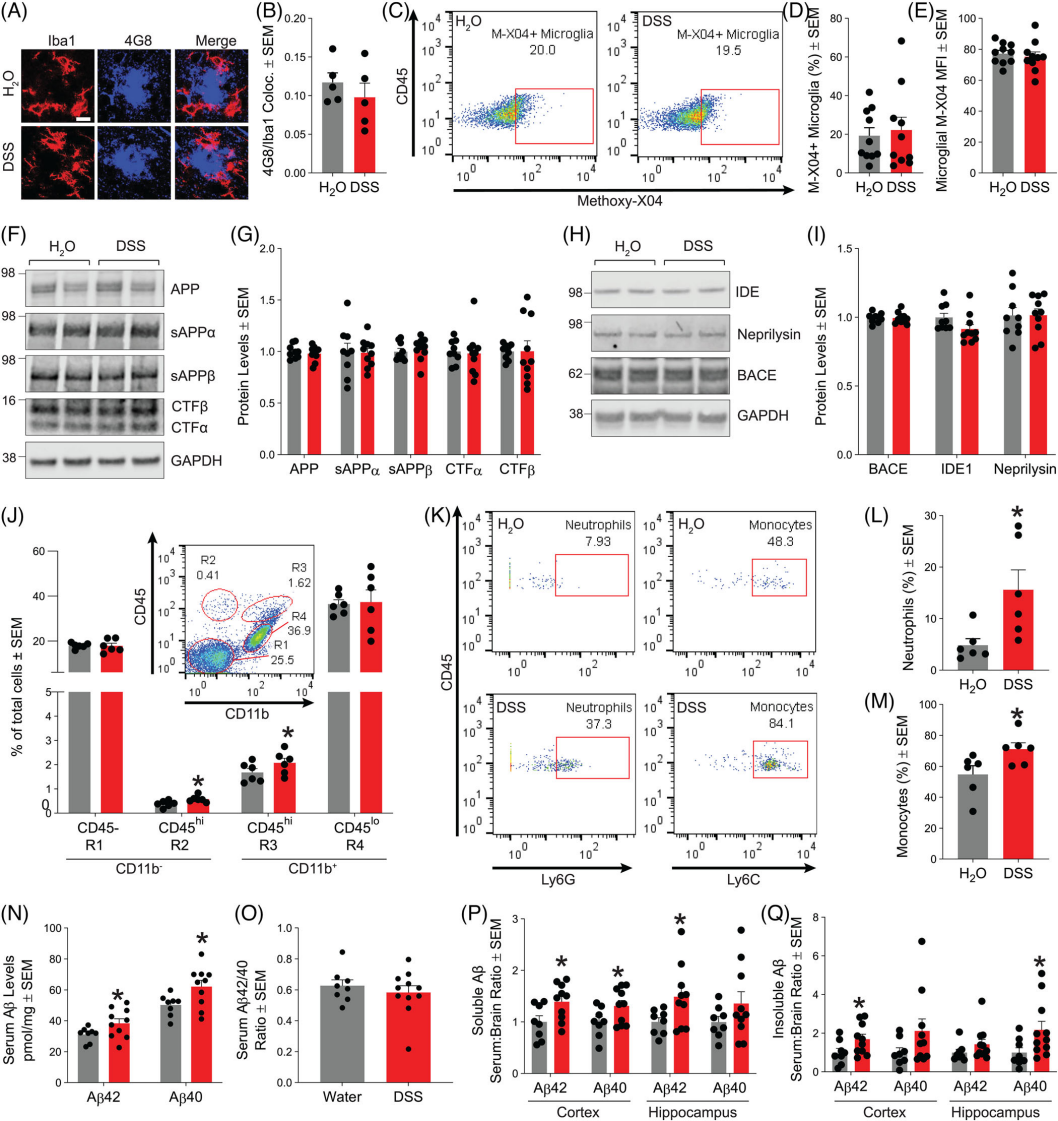
Leukocyte infiltration and increased serum Aβ present during acute colitis in 5xFAD mice. (A) Representative images from mouse subiculum stained for 4G8 and Iba1 to detect colocalization. Scale bar = 12 µm. (B) Mander's M1 colocalization coefficient between 4G8 and Iba1. Statistical analysis was carried out by a two‐tailed Student's *t*‐test (*n* = 5 mice/group). (C) Flow cytometry scatter plot from CD11b+‐CD45low gated whole brain microglia positive for methoxy‐X04 on a control 5xFAD mouse that did not receive an intraperitoneal methoxy‐X04 injection. (D) Percent population of gated CD11b+‐CD45low gated whole brain microglia positive for methoxy‐X04 (*n* = 10 mice/group from two independent experiments). (E) Mean methoxy‐X04 fluorescence of gated CD11b+‐CD45low gated whole brain microglia positive for methoxy‐X04 (*n* = 10 mice/group from two independent experiments). (F) Representative Western blots from mouse cortical homogenates probed for total APP, sAPPα, sAPPα, C‐terminal APP fragments and GAPDH. (G) Quantification of total APP, sAPPα, sAPPα, and C‐terminal APP fragments normalized to GAPDH (*n* = 9–10 mice/group from two independent experiments). (H) Representative Western blots from mouse cortical homogenates probed for IDE, neprilysin, BACE and GAPDH. (I) Quantification of IDE, neprilysin, and BACE normalized to GAPDH (*n* = 9–10 mice/group from two independent experiments). (J) Flow cytometry scatter plots showing the percentage of total cells in mouse brain single cell suspensions stained for CD45 and CD11b (*n* = 6 mice/group, *p < 0.05). (K) Flow cytometry scatter plots of mouse brain single cell suspensions positive for Ly6G and Ly6C staining after initial gating of the CD11b+CD45+ cell population. (L) Percentage of neutrophils (CD11b+CD45+Ly6G+) and monocytes (M) (CD11b+CD45+Ly6C+) in mouse brain single cell suspensions. Statistical analyses were performed by either two‐tailed unpaired *t*‐tests, one or two‐way ANOVAs with Bonferroni post‐hoc tests. (N) Soluble Aβ42 and Aβ40 levels in mouse serum (n = 8‐10 mice/group from two independent experiments, *p < 0.05). (O) Average soluble Aβ42/40 ratios in mouse serum (*n* = 8–10 mice/group, *p < 0.05, **p < 0.01). (P) Corresponding serum to brain ratio of either RIPA soluble Aβ42 or Aβ40 levels in the cortex and hippocampus (*n* = 8–10 mice/group from two independent experiments, *p < 0.05). (Q) Corresponding serum to brain ratio of either formic acid (FA) soluble Aβ42 or Aβ40 levels in the cortex and hippocampus (*n* = 8–10 mice/group from two independent experiments, *p < 0.05). Statistical analyses were carried out by two‐tailed unpaired *t*‐tests. ANOVA, analysis of variance; APP, amyloid precursor protein; GAPDH, glyceraldehyde‐3‐phosphate dehydrogenase.

To understand the mechanism behind the reduction in beta‐amyloid deposition we observed as a result of acute colitis, we first performed immunofluorescence of microglia (Iba1) and beta‐amyloid (4G8) in the subiculum of brain tissue sections (Figure 5A) and measured colocalization as a measure of microglial phagocytosis. Surprisingly, no changes in colocalization were observed following DSS treatment (Figure 5B). To further corroborate this finding, we performed microglial internalization of methoxy‐X04 labeled beta‐amyloid using flow cytometry (Figure 5C). Similarly, we did not find changes in the internalization of beta‐amyloid (Figure 5D–E). Further, APP processing (Figure 5F–G) or the levels of BACE1 and beta‐amyloid degrading enzymes (Figure 5H–I) were altered by acute colitis.

Given the endothelial activation we observed in the brain following acute colitis, we decided to explore whether leukocyte infiltration occurred in our acute colitis model, since monocytes, for example, can phagocytose beta‐amyloid.^31^, ^32^, ^33^ Through flow cytometry of brain single‐cell suspensions, we observed increased peripheral cell infiltration, namely lymphocytes (CD45^hi^CD11b^−^) and leukocytes (CD45^hi^CD11b^+^), into the brain (Figure 5J). We further characterized the leukocyte population and determined that acute colitis led to brain infiltration of neutrophils (CD45^hi^CD11b^+^Ly6G^+^; Figure 5K–L) and monocytes (CD45^hi^CD11b^+^Ly6C^+^; Figure 5K and 5M ). Additionally, we confirmed the presence of monocytes in the brain, particularly around the highly vascularized dentate gyrus, through immunofluorescence (Figure S7A, B) using the monocyte marker F4/80. We were also able to detect monocytes around blood vessels of the dentate gyrus using chromogenic (3,3'‐diaminobenzidine [DAB]) immunohistochemistry (Figure S7C). We surmise that these monocytes are not microglia due to their low colocalization with Iba1 (data not shown) as well as the absence of F4/80 immunofluorescence in the subiculum, a region with increased microgliosis as seen in Figure 3B.

While leukocyte infiltration, particularly monocytes, could be associated with the reduction in brain beta‐amyloid, we also decided to measure serum soluble Aβ42 and Aβ 40 since beta‐amyloid can be exported (or cleared) through the BBB. While we observed decreased beta‐amyloid deposition and insoluble accumulation in the brain, serum Aβ42 and Aβ40 were significantly elevated by acute colitis (Figure 5N). No difference in the Aβ42/40 ratio was observed in the serum (Figure 5O). To dissect the relationship between brain and serum Aβ42 and Aβ40, we calculated a serum‐to‐brain ratio by dividing the respective serum and brain (soluble or insoluble) Aβ42 and Aβ40 concentrations from individual mice. The serum‐to‐brain ratio using soluble brain Aβ42 and Aβ40 levels was significantly increased in the acute colitis group compared to the control (Figure 5P). A similar correlation was observed in the serum‐to‐brain ratio using insoluble (FA soluble) Aβ42 and Aβ40 levels (Figure 5Q). The beta‐amyloid serum‐to‐brain ratio calculations, therefore, suggested increased beta‐amyloid efflux from the brain into the bloodstream.

## 4 DISCUSSION

Emerging evidence supports a strong link between the gut microbiome and AD pathogenesis. A recent study by Zhang et al.^34^ showed that patients with inflammatory bowel disease (IBD) have a higher incidence of dementia compared to controls, underscoring the need to better understand the role of the gut‐brain axis in the pathogenesis of AD and other neurodegenerative diseases. Here, we explored the effects of acute intestinal inflammation (colitis) on AD pathology, including brain neuroinflammation and beta‐amyloid deposition, using the DSS colitis model (acute colitis) in 5xFAD mice. We determined that acute colitis robustly altered colonic architecture, altered microbial diversity, and induced systemic inflammation. Acute colitis led to brain endothelial activation followed by microglial activation, the release of pro‐inflammatory cytokines, and infiltration of neutrophils and monocytes. Surprisingly, these changes in response to acute colitis led to a significant reduction in beta‐amyloid deposition in the brain concomitant with increased serum beta‐amyloid levels.

We showed that microglial phagocytosis was not responsible for the reduced beta‐amyloid deposition nor was APP processing significantly altered following acute colitis. Mezö^35^ showed that while both germ‐free and antibiotic‐treated 5xFAD mice presented with reduced beta‐amyloid deposition, germ‐free mice displayed increased microglial phagocytosis and antibiotic‐treated mice did not, suggesting dysbiosis as opposed to the absence of a microbiome have distinct effects on how beta‐amyloid is processed. One hypothesis for how acute colitis affects beta‐amyloid deposition is that the infiltration of peripheral cells in DSS‐treated mice could phagocytose beta‐amyloid, as a study reported that recruited monocytes are approximately three times more phagocytic than microglia and can effectively clear beta‐amyloid.^31^, ^32^, ^33^ It has also been reported that a perturbed gut microbiome can lead monocytes to exhibit increased phagocytic and anti‐microbial abilities.^36^ In fact, monocyte ablation in AD transgenic mice led to the accumulation of brain beta‐amyloid and enrichment ameliorated this effect, highlighting the importance of monocyte infiltration.^37^, ^38^ Increased circulating endotoxins in 5xFAD mice subjected to acute colitis may serve to expand the monocyte population, which can function as immune surveillance in clearing threats or debris, including beta‐amyloid in the brain. Leukocyte extravasation into the brain in our model could occur as a result of BBB breakdown or through transendothelial migration.^39^ Nevertheless, we only found an association between monocyte infiltration and reduced deposition of beta‐amyloid, so future studies are needed to understand whether intestinal inflammation can promote monocyte‐dependent phagocytosis of beta‐amyloid. Cytokines released are important regulators of cellular function that can impact cognitive function^40^, ^41^ and play a role in priming innate immune cells to fight acute inflammation and infection.^42^ Thus, their expression levels in the brain as well as bloodstream could likely be involved in regulating beta‐amyloid levels in our study as well as the phagocytic activity of leukocytes.

We and others,^43^, ^44^ found that acute colitis impacts microbial diversity and modifies the microbiome similar to antibiotic treatments. Acute colitis induces dysbiosis through intestinal inflammation, since DSS in the absence of a host does not impact microbial diversity.^45^ Interestingly, studies disrupting the gut microbiome with antibiotic cocktails^46^, ^47^, ^48^ or through the generation of germ‐free beta‐amyloid overexpressing mice,^49^ all reported decreased cerebral beta‐amyloid pathology similar to our study using DSS‐induced colitis. Considering that DSS can breach the intestinal epithelial barrier and increase serum endotoxins,^50^, ^51^ the observed reduction in beta‐amyloid pathology following acute DSS treatment is consistent with several reports indicating that lipopolysaccharide (LPS) treatments, either systemic^52^ or intracranial,^53^, ^54^, ^55^ lead to reduced cerebral beta‐amyloid deposition. These results suggest an intimate relationship between beta‐amyloid levels and gut microbiota modulation. Further strengthening a link between intestinal inflammation (microbiome modulation) and beta‐amyloid levels, models of colitis, such as IL‐10^56^, ^57^ and RAG2^58^ knockouts (KO), when crossed with beta‐amyloid overexpressing mice, show a reduction in brain beta‐amyloid deposition (though the impact of colitis in their mouse model studies was not directly tested).^59^, ^60^ The exact mechanisms underlying these effects warrant further research.

Here, we observed that the decrease in beta‐amyloid levels following acute colitis also correlated with increased export of beta‐amyloid into the blood. In light of the emerging role of beta‐amyloid as an AMP,^7^, ^61^, ^62^ the release of brain beta‐amyloid by the brain could be in response to the presence of circulating endotoxins due to a breach of the gut epithelial barrier by DSS. Immunodeficient RAG2 KO crossed with APP‐PS1, for instance, also show increased serum beta‐amyloid,^58^ perhaps as a protective mechanism. Therefore, it is likely that constant efflux of beta amyloid into the blood circulation over the course of DSS treatment might be enough to effect pronounced reductions in beta‐amyloid in young 5xFAD mice without extensive beta‐amyloid deposition. Future studies are needed to understand the underpinnings behind brain beta‐amyloid release into the bloodstream during acute colitis. A major candidate pathway is the P‐gp‐LRP1‐PICALM complex in endothelial cells that is known to be involved in the systemic efflux of beta‐amyloid.^63^, ^64^, ^65^ It is also possible that impaired beta‐amyloid influx into the brain following acute colitis, possibly through RAGE,^66^ might also be involved in keeping serum beta‐amyloid levels high, in order to retain the AMP in circulation.

Of note is current research showing that chronic colitis treatment using DSS in 10‐month‐old AppNL‐G‐F mice led to increased beta‐amyloid deposition,^67^ seemingly contradicting our findings. However, our study used 2‐month‐old mice and a seven‐day acute DSS treatment. Hence, the discrepancies could be due to age‐related changes in the expression of proteins responsible for the beta‐amyloid influx and efflux^68^, ^69^, ^70^ through the BBB, leading to increased beta‐amyloid aggregation. Additionally, changes in monocyte phenotype and phagocytic activity with age could account for the differences. Changes in the microbiome and intestinal integrity in older mice, as well as the response to DSS, are likely factors as well. Therefore, while the results of the aforementioned chronic colitis study might be due to the aging milieu, our results might portray an acute physiological response to systemic inflammation prior to substantial beta‐amyloid plaque deposition, behavioral deficits, as well as aging‐related changes in brain structure and function. It is also important to mention that there could be differences in beta‐amyloid responsiveness between acute and chronic colitis, for example, microglial and monocyte homeostasis, as well as cytokine release. Further, it has been shown that early neuroinflammation in cognitively normal older adults is associated with lower beta‐amyloid levels,^71^ highlighting the usefulness of our acute model to assess the early dynamics of disease‐associated proteins, such as beta‐amyloid, prior to significant accumulation and cognitive decline.

In conclusion, here we report that acute colitis in young 5xFAD mice leads to unique alterations, including reduced brain beta‐amyloid deposition and increased efflux into the blood circulation, that are associated with typical features in colitis models such as dysbiosis, elevated serum endotoxins and pro‐inflammatory cytokines, enhanced infiltration of leukocytes into the brain, and microglial activation. While it is possible that acute intestinal inflammation could inadvertently promote beta‐amyloid degradation through various means (i.e., phagocytosis), these results, along with the increased relevance of beta‐amyloid in microbial entrapment,^7^, ^61^, ^62^ highlight the responsiveness of beta‐amyloid to systemic inflammation and infection that could be altered with age and disease. It is important to note that DSS‐induced colitis does not recapitulate the development of colitis (as it is driven chemically), therefore, other relevant models of intestinal inflammation and dysbiosis, such as antibiotic‐induced or through fecal microbiota transplantation, will be useful to corroborate our findings. Also, given the diverse impacts of colitis on different models of beta‐amyloid position, it would be of interest to generate comparative studies across these models. Most importantly, future research is needed not only to understand the function of beta‐amyloid, but also to investigate its dynamics when the intestinal microbiome is altered. For example, in germ‐free mice as well as in response to dysbiosis linked to systemic inflammation, intestinal inflammation, and antibiotic treatments.

## CONFLICT OF INTERESTS STATEMENT

R.E.T. has equity in Marvel Biome and serves on their scientific advisory board. All other authors have no conflicts. Author disclosures are available in the Supporting Information.

## CONSENT STATEMENT

All human subjects provided informed consent, or consent was unnecessary.

## Supporting information



Supporting Information

Supporting Information
