# Supplementary material for: Acute experimental colitis in 5xFAD Alzheimer's disease mice leads to enhanced monocyte infiltration into the brain accompanied by reduced β‐amyloid deposition
Source: Alzheimers Dement. 2025 Jun 2;21(6):e70292. doi: 10.1002/alz.70292 (PMC12130571; doi:10.1002/alz.70292)
Supplement: Supplementary file 2 — Supporting Information [file ALZ-21-e70292-s002.pdf]

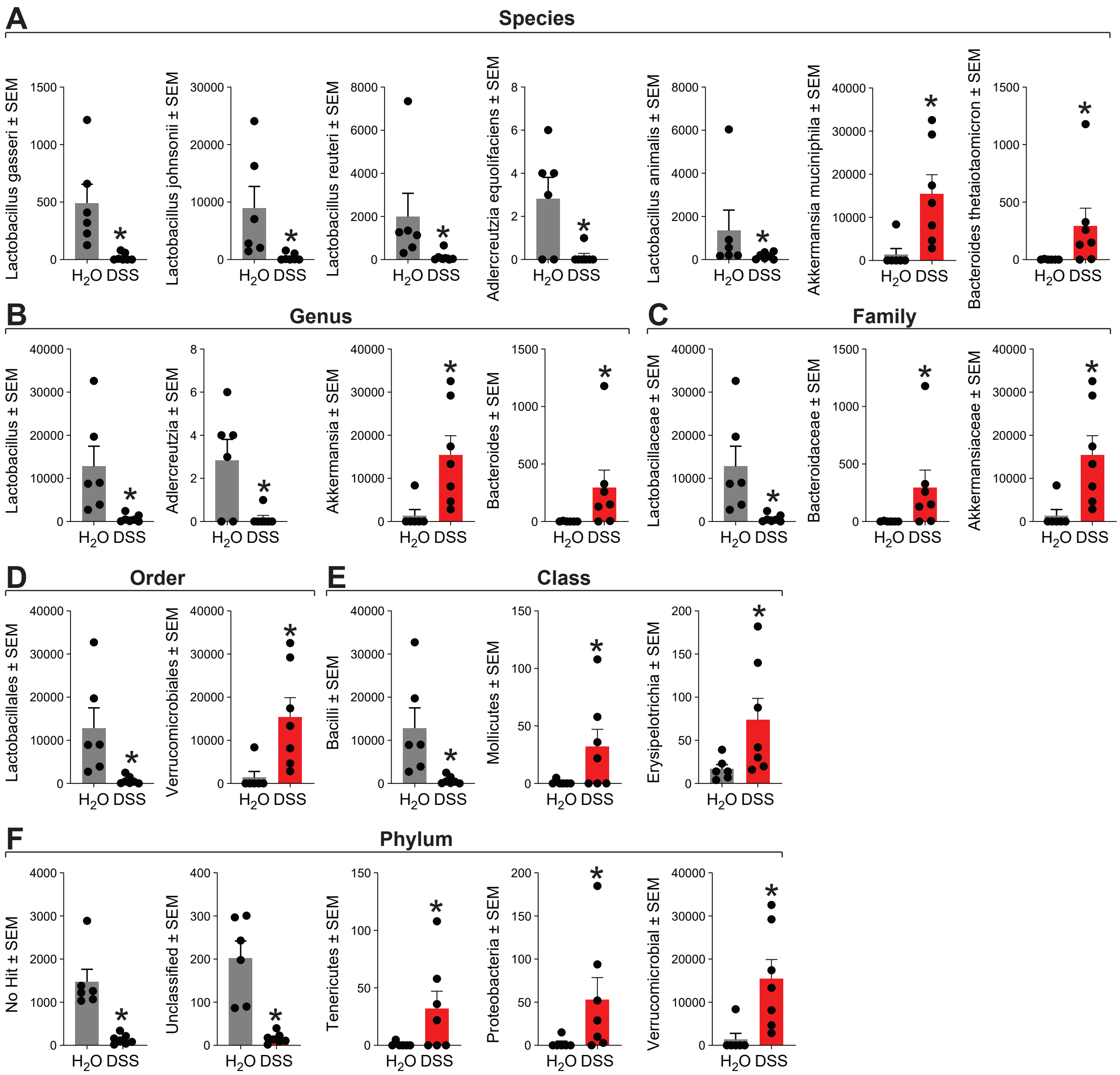

Supplementary Figure 1. Acute colitis affects taxa in 5xFAD stool samples. (A) Significantly differences species in stool samples (n = 6-7/group, * $p < 0.05$). **(B)** Significantly differences genera in stool samples (n = 6-7/group, * $p < 0.05$). **(C)** Significantly differences families in stool samples (n = 6-7/group, * $p < 0.05$). **(D)** Significantly differences orders in stool samples (n = 6-7/group, * $p < 0.05$). **(E)** Significantly differences classes in stool samples (n = 6-7/group, * $p < 0.05$). **(F)** Significantly differences phyla in stool samples (n = 6-7/group, * $p < 0.05$). **(G)** Significantly differences species in stool samples (n = 6-7/group, * $p < 0.05$).

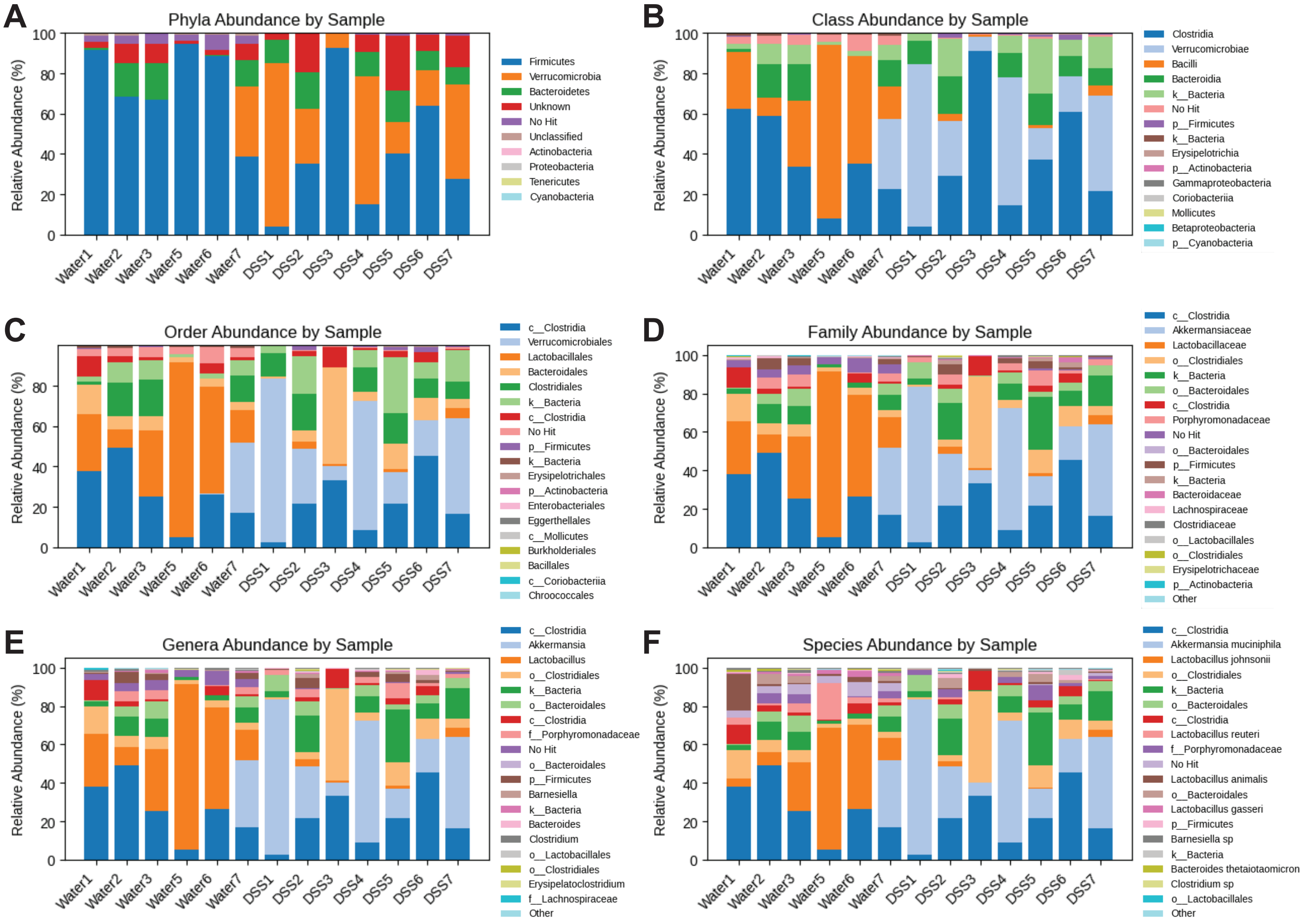

Supplementary Figure 2. Taxa in individual control and Acute colitis 5xFAD stool samples. (A) Phyla per individual mouse stool samples. **(B)** Classes per individual mouse stool samples. **(C)** Orders per individual mouse stool samples. **(D)** Families per individual mouse stool samples. **(E)** Genera per individual mouse stool samples. **(F)** Species per individual mouse stool samples.

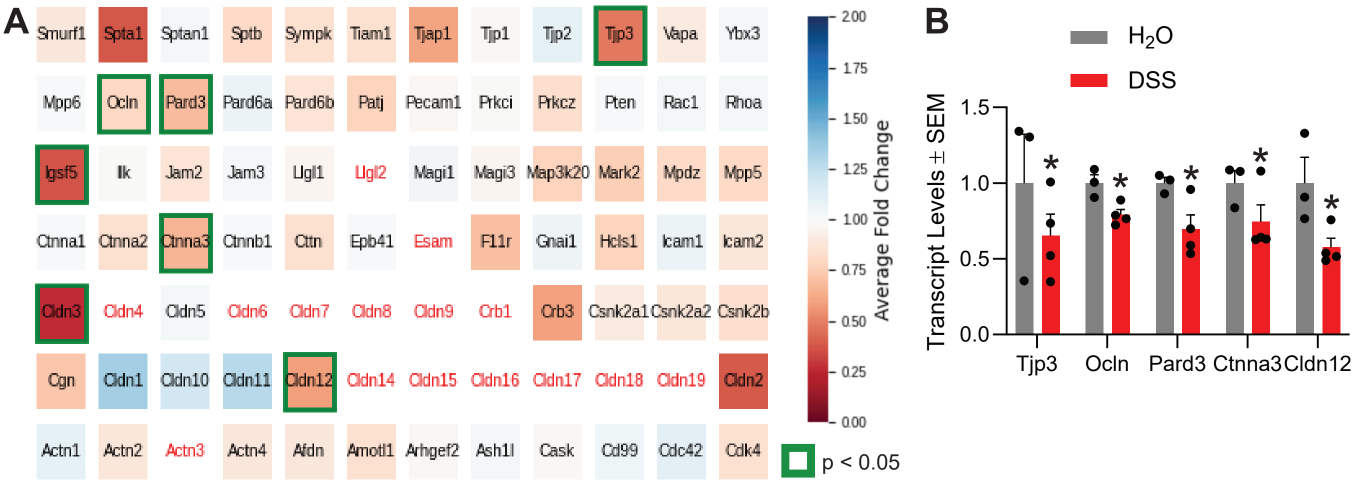

Supplementary Figure 3. Acute colitis impacts blood-brain barrier tight junction transcripts. (A) Heat map of 84 gene transcripts measured in mouse brain cortical RNA samples (n = 3-4/group). Transcripts in red font were not detectable in our samples. Transcripts with a green border have significantly altered expression ($p < 0.05$). **(B)** Bar graphs of significantly altered transcripts (* $p < 0.05$).

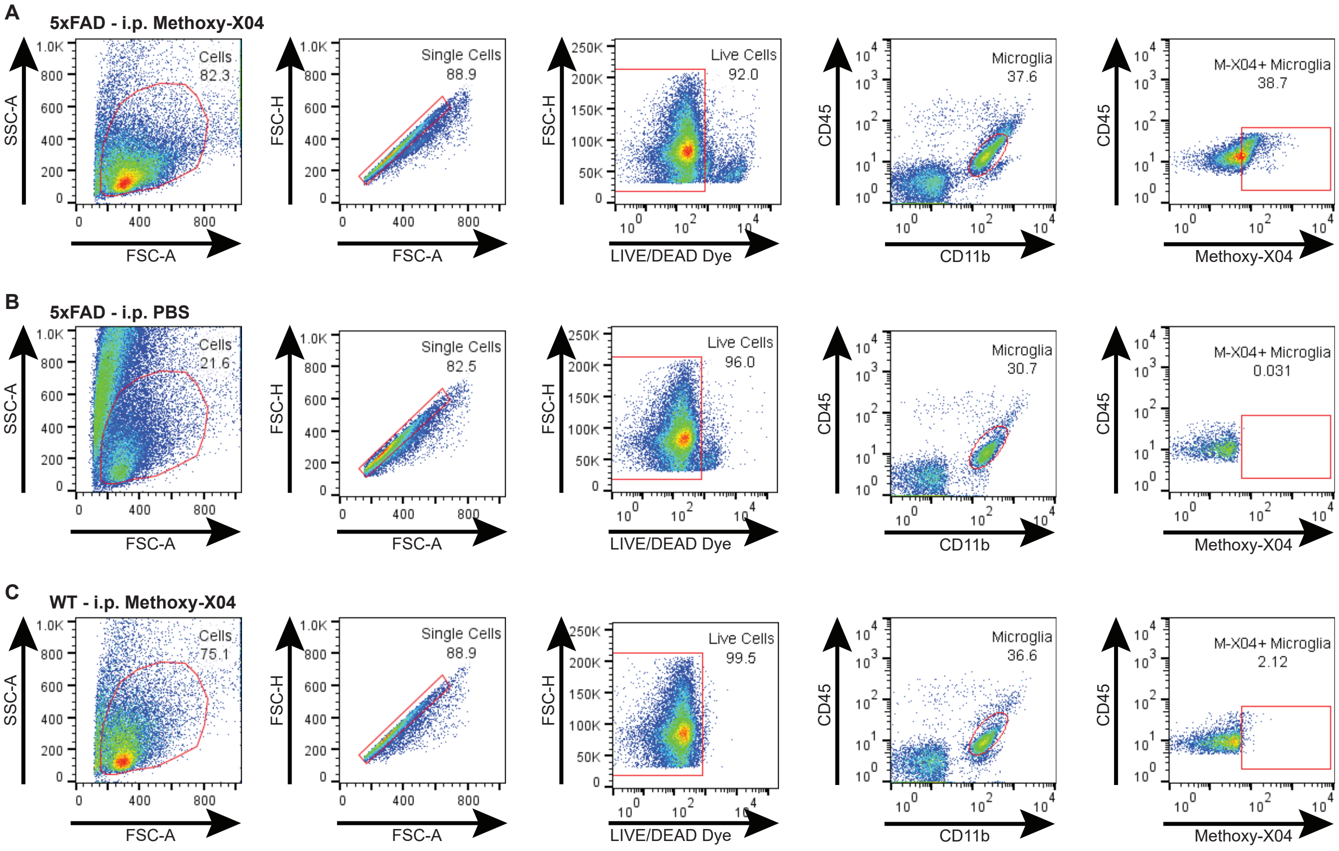

Supplementary Figure 4. Microglia gating strategy from mouse brain single cell suspensions. To identify microglia in mouse brain single cell suspensions, cells were first identified on a scatter plot with the forward and side scatters. Then cells were plotted against the amplitude and height forward scatters to identify single cells. From then, live single cells were gated based on the lack of LIVE/DEAD dye fluorescence. Finally, live single cells were plotted against the CD11b and CD45 markers to identify the CD45-low, CD11b high (+) population, which are microglia. Methoxy X04 fluorescence can then be measured within the microglial population. Representative gating strategy scatter plots for **(A)** a 5xFAD mouse that received an intraperitoneal (i.p.) methoxy X04 injection, **(B)** a 5xFAD mouse that received an i.p. PBS injection, and **(C)** a wild-type mouse that received an i.p. methoxy X04 injection.

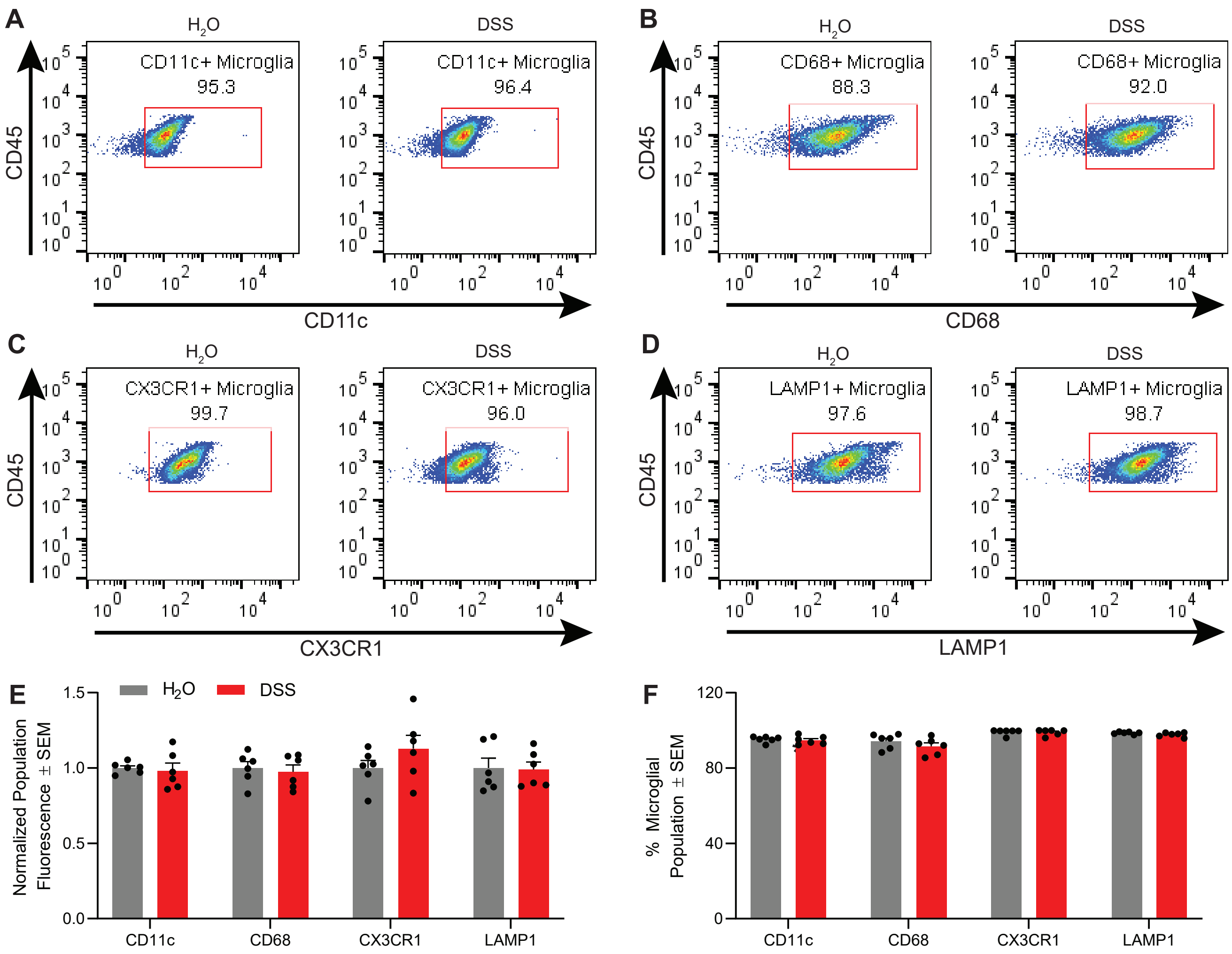

Supplementary Figure 5. Acute colitis does not alter CD11c, CD68, CX3CR1, or LAMP1 expression in 5xFAD microglia. Scatter plots for **(A)** gated microglia positive for the CD11c surface marker, **(B)** gated microglia positive for the CD68 marker. **(C)** Gated microglia positive for the CX3CR1 surface marker, and **(D)** gated microglia positive for the LAMP1 marker. **(E)** Mean fluorescence of CD11c, CD68, CX3CR1, and LAMP1 in their corresponding positive microglia population (n = 6 mice/group, * $p < 0.05$). **(F)** Percent population of total microglia positive for CD11c, CD68, CX3CR1, and LAMP1 (n = 6 mice/group, * $p < 0.05$). Statistical analyses were performed by two-tailed unpaired t-tests.

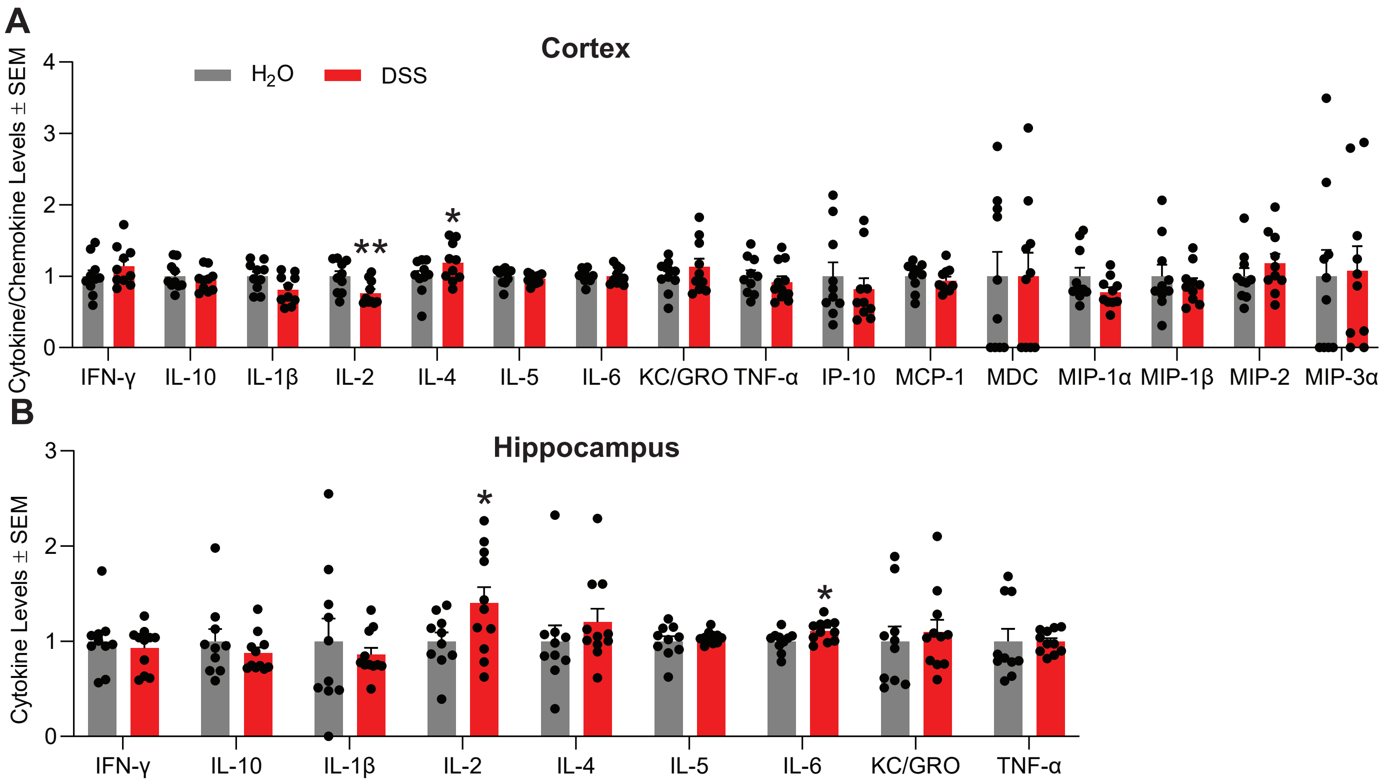

Supplementary Figure 6. Acute colitis increases pro-inflammatory cytokines in the brain of 5xFAD mice. (A) Concentration of pro-inflammatory cytokines in mouse cortical homogenates (n = 10-11 mice/group from two independent experiments, * $p < 0.05$, ** $p < 0.01$). **(B)** Concentration of pro-inflammatory cytokines in mouse hippocampal homogenates (n = 10-11 mice/group from two independent experiments: * $p < 0.05$, ** $p < 0.01$). Statistical analyses were performed by two-tailed unpaired t-tests.

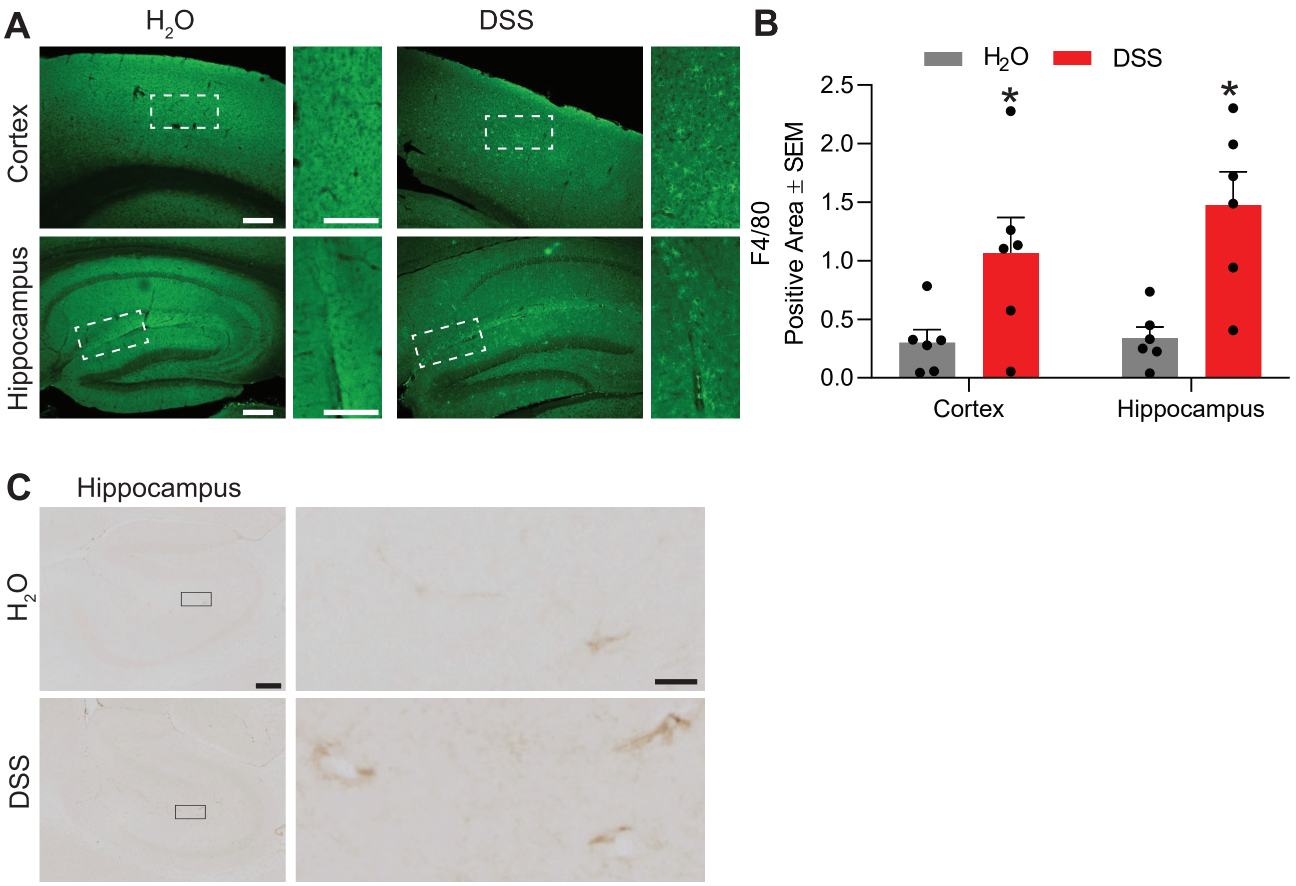

Supplementary Figure 7. Acute colitis increases F4/80+ monocytes in the 5xFAD brain. (A) Representative images of the cortex and hippocampus stained for F4/80. Scale bar = 200 and 20 μm. **(B)** Quantification of percent positive area for F4/80 in the cortex and hippocampus (n = 6 mice/group, * $p < 0.05$). Statistical analyses were performed by two-tailed unpaired t-tests. **(C)** Representative images of the hippocampus staining for F4/80 using chromogenic DAB immunohistochemistry.
